# CARE-Net: A Compact Framework for Vibration Damper Detection in UAV-Based Transmission Line Inspection

**DOI:** 10.3390/s26175648

**Published:** 2026-09-05

**Authors:** Yujie Zhou, Chao Ji, Huan Wang, Long Zhao, Peng Yang, Chao Zhang

**Affiliations:** 1 School of Electronics and Information, Xi’an Polytechnic University, Xi’an 710048, China; 13210207067@163.com (Y.Z.); wh15229983532@163.com (H.W.); zhaolong@xpu.edu.cn (L.Z.); 2 China National Heavy Machinery Research Institute Co., Ltd., Xi’an 710016, China18455094050@163.com (C.Z.)

**Keywords:** transmission line inspection, vibration damper condition detection, slender small-object detection, compact detection framework, CARE-Net

## Abstract

Vibration damper detection in unmanned aerial vehicle (UAV)-based transmission line inspection presents distinctive task-specific challenges: the targets are not only small and weakly textured, but also characterized by slender structures. Their effective identification therefore depends on the preservation of local contour cues and the appropriate organization of deep contextual responses. To address the limitations of conventional lightweight detectors in structural feature representation, cross-scale semantic consistency, and bounding-box localization, this paper proposes CARE-Net (Cascaded Attention and Refinement Enhanced Network), a compact detection framework for vibration damper detection. CARE-Net adopts an asymmetric design consisting of front-end structural enhancement and back-end contextual refinement. Specifically, the Cascaded Residual Attention Block (CRAB) is deployed in the backbone to strengthen the representation of slender contours and local structural features of vibration damper targets. The Dynamic Context Refinement Network (DCRN) is introduced at the backbone–neck transition to improve the contextual organization of deep features and the quality of cross-scale feature fusion. Meanwhile, an Adaptive Focal Complete IoU Loss (AF-CIoU) is proposed to optimize bounding-box regression for difficult samples without altering the inference architecture. A UAV-based vibration damper dataset covering three condition categories, namely normal, rusted, and dilapidated, is constructed in this study. Experimental results show that CARE-Net achieves an mAP^@0.5^ of 0.951 and an mAP^@0.5:0.95^ of 0.628 with 2.44 M parameters and 6.2 GFLOPs. Further configuration experiments indicate that, compared with repeatedly introducing attention enhancement into high-level features, stage-specific feature modeling is better suited to the slender small-object detection task investigated in this study. The proposed method provides a solution for intelligent vibration damper inspection of transmission lines that balances detection accuracy, model compactness, and potential for terminal-side application.

## 1. Introduction

With the continuous expansion of power systems and the increasing number of transmission lines, condition monitoring of line fittings has become an essential task for ensuring the safe and stable operation of power grids [1,2]. As a critical fitting installed on overhead transmission lines, the vibration damper is designed to suppress wind-induced conductor vibration, mitigate fatigue damage, and extend conductor service life. Its installation condition and structural integrity are therefore closely related to the long-term operational reliability of transmission lines [3]. During prolonged operation, vibration damper components may be affected by wind-induced vibration, rain and snow erosion, material aging, installation displacement, and other environmental disturbances, resulting in abnormal conditions such as loosening, corrosion, displacement, and structural damage. If these defects are not identified in a timely manner, they may accelerate conductor fatigue accumulation and, in severe cases, cause strand breakage, fitting detachment, or transmission failures, thereby posing potential risks to power grid operation [4]. Consequently, efficient, accurate, and stable vibration damper detection holds substantial engineering value for transmission line inspection.

In recent years, UAV-based visual inspection has increasingly become an effective alternative to conventional manual inspection. Compared with tower-climbing inspection and ground-based visual observation, UAV inspection offers greater mobility, broader coverage, lower operational risk, and higher data acquisition efficiency, allowing transmission line images to be rapidly collected in complex terrain, long-distance transmission corridors, and high-altitude operating environments [5]. Nevertheless, vibration damper targets in practical UAV inspection imagery typically exhibit small-scale and slender structural characteristics, together with weak surface texture and low grayscale contrast. Their visual appearance may be highly similar to conductors, insulator strings, tower fittings, vegetation, sky regions, and other complex backgrounds. Moreover, long-distance imaging, uneven illumination, haze, rain and snow, partial occlusion, and background interference may further weaken edge and local structural cues, making vibration damper detection susceptible to missed detections, false detections, and localization errors [6,7].

With the development of deep learning, object detection methods have been widely applied to transmission line inspection and small-object recognition. Existing related studies can be broadly categorized into three groups. The first group focuses on detection architecture enhancement, in which network structures, feature extraction units, or detection heads are modified to improve small-object representation. For example, Wu et al. [8] proposed SOD-YOLO, which introduces an additional small-object detection layer and adjusts the multi-branch structure within the YOLOv8n framework, thereby improving the detection performance of small-scale components, including vibration damper targets. Song et al. [9] proposed Deformable-YOLOX, which integrates deformable convolution, a decoupled detection head, and adaptive spatial feature fusion (ASFF) to enhance fine-grained semantic representation for densely distributed line fittings, demonstrating the effectiveness of structural redesign in transmission line component detection.

The second group investigates structural priors and domain-generalized modeling, aiming to improve cross-scene detection stability by introducing geometric structural constraints, morphological statistics, or domain-invariant feature representations. Yang et al. [10] proposed the One-for-All framework, which combines structural priors, multi-scale geometric representation, and domain-invariant feature extraction to enhance the generalization capability of vibration damper detection across different inspection scenarios. Zhao et al. [11] developed a structural knowledge representation model that improves intra-class discrimination by modeling morphological statistics and appearance proportion relationships of vibration damper targets. Liu et al. [12] improved a detection network by integrating ResNet and PANet, using deep feature fusion to enhance vibration damper recognition performance, which indicates the importance of multi-scale deep feature integration for small fitting detection. Wu et al. [13] further proposed Cognition-Driven DDDNet, which combines cognitive preprocessing with heterogeneous feature pyramid fusion and achieves competitive detection performance on the Damper-DET dataset.

The third group addresses robust detection under complex environments and degraded imaging conditions. Ji et al. [14] proposed STAE-YOLO, which exploits the cross-scale semantic modeling capability of Swin Transformer to improve target recognition in complex inspection scenes. Jia et al. [15] developed AdaptoMixNet, which employs adaptive feature mixing and an enhanced feature pyramid to improve detection stability under conditions such as fog and backlighting. To address rain-induced image degradation, Ji et al. [16] proposed DRI-Net, which integrates a DRGAN-based deraining module with a detection network to achieve robust insulator defect detection in rainy environments. Although its target object differs from that considered in vibration damper detection, this work illustrates the importance of enhanced visual representation for transmission line component detection under degraded imaging conditions. Collectively, these studies indicate that strengthening semantic modeling, improving multi-scale feature fusion, and enhancing adaptability to complex environments are important directions for advancing small-object detection in transmission line inspection.

Despite the progress achieved in recent studies, the task-specific challenges encountered in UAV-based vibration damper inspection remain insufficiently addressed. For slender and weak-texture vibration damper targets, relying solely on local convolution or fixed-scale feature fusion may produce discontinuous structural responses. Fine-grained information associated with target edges, damper heads, and connection regions may gradually diminish during deep feature propagation, resulting in fragmented feature representations and incomplete semantic expression. Meanwhile, long-distance imaging and complex backgrounds may further amplify response discrepancies among features at different scales. Semantic redundancy may arise in high-level pyramid features, whereas low-level detailed features remain vulnerable to background texture interference, eventually leading to semantic degradation and scale mismatch during cross-scale feature fusion. In addition, although existing lightweight detectors are generally advantageous in terms of parameter count and inference speed, they remain susceptible to false detections and missed detections in scenes involving occlusion, low contrast, and strong background similarity. Recent studies have also explored uncertainty-aware knowledge transfer to improve the accuracy–efficiency balance of lightweight transmission-line inspection models, indicating that effective information selection is important for maintaining detection performance under compact model constraints [17]. Meanwhile, localization-oriented learning has received increasing attention in object detection. Zheng et al. [18] demonstrated the importance of explicitly transferring localization knowledge for dense detectors, while Li et al. [19] incorporated localization quality into dense detection optimization through quality-aware bounding-box modeling. For small-scale vibration damper targets with pronounced aspect-ratio variations, conventional IoU-based losses may also be insufficiently responsive to bounding-box displacement, scale variation, and difficult sample distributions, thereby limiting regression stability and localization accuracy under high IoU thresholds.

To address these issues, this paper proposes CARE-Net (Cascaded Attention and Refinement Enhanced Network), a compact detection framework for vibration damper detection. We argue that, for slender and weak-texture targets in UAV inspection imagery, improving detection performance requires more than enhanced local feature extraction; it also requires coordinated modeling of structural cue preservation, deep contextual organization, and localization optimization. Based on this consideration, CARE-Net adopts an asymmetric design comprising front-end structural enhancement and back-end contextual refinement. Specifically, the Cascaded Residual Attention Block (CRAB) is deployed in the backbone to preserve discriminative structural cues associated with contours, edges, and connection regions of vibration damper targets. The Dynamic Context Refinement Network (DCRN) is introduced at the backbone–neck transition to dynamically refine deep contextual representations before multi-scale feature fusion, thereby alleviating semantic interference and scale inconsistency in complex backgrounds. On this enhanced feature basis, the Adaptive Focal Complete IoU Loss (AF-CIoU) is incorporated to perform quality-aware optimization of bounding-box regression, enabling localization constraints to act more effectively on difficult predictions of slender small objects. This design mitigates potential representational redundancy caused by indiscriminate repeated enhancement of high-level semantic features and establishes a stage-specific coordinated optimization framework for vibration damper detection under compact model constraints.

The main contributions of this paper are summarized as follows:

(1) A compact vibration damper detection framework, termed CARE-Net, is proposed for UAV-based transmission line inspection. It is specifically designed to address the small scale, slender structure, weak texture, and pronounced background interference associated with vibration damper targets, improving detection across three condition categories, namely normal, rusted, and dilapidated, while maintaining low model complexity.
(2) A stage-specific coordinated optimization mechanism is developed for slender small-object detection. CRAB enhances local structural and contour representations in the backbone, DCRN dynamically refines contextual responses before deep feature fusion, and AF-CIoU further improves bounding-box localization stability through quality-aware regression weighting. Together, these components establish an effective connection among structural representation, contextual modeling, and localization optimization.
(3) Comparative experiments, component ablation studies, deployment-position analyses, and visualization-based application demonstrations are conducted to evaluate CARE-Net for vibration damper detection. The results show that the proposed method achieves a favorable balance between detection accuracy and model complexity, while exhibiting potential for integration into terminal-side analysis workflows for intelligent transmission line inspection.

## 2. Proposed Method

### 2.1. Transmission Line Vibration Damper Detection System

#### 2.1.1. Overall System Architecture

**Figure 1:**
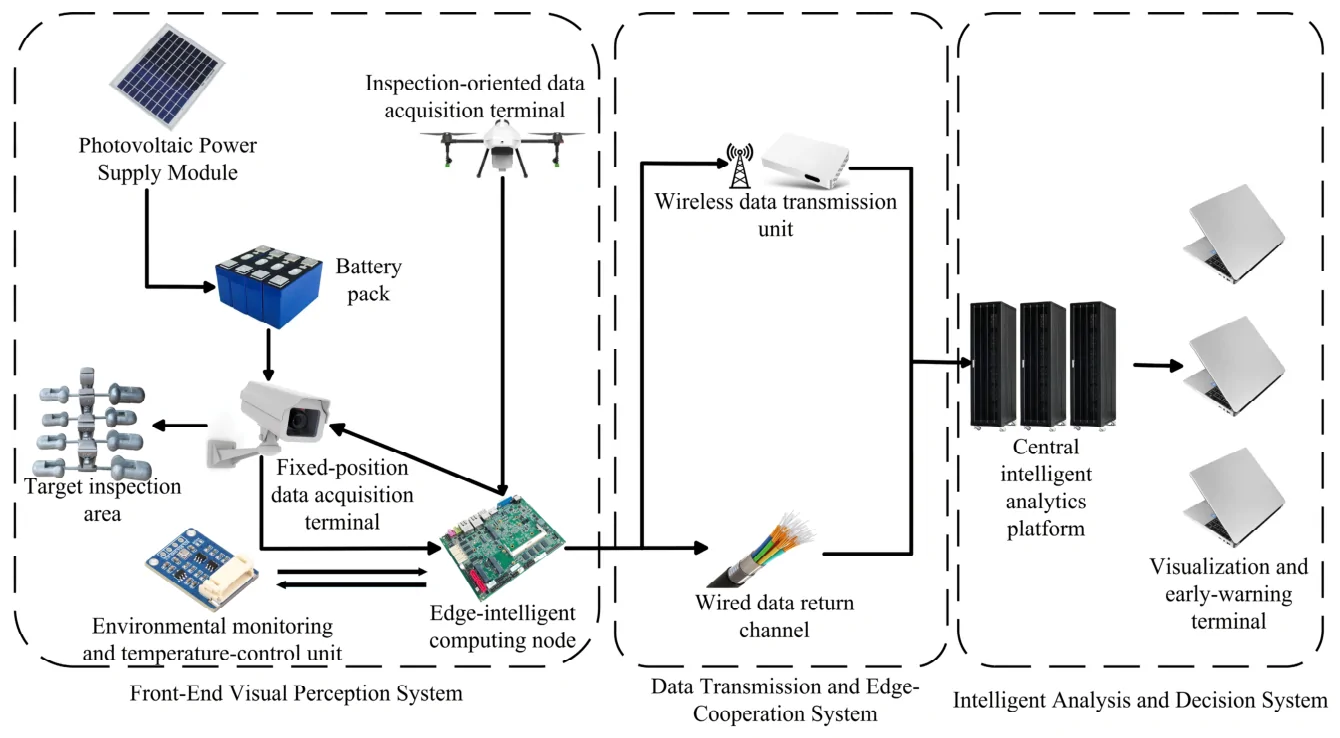
Overall architecture of the transmission-line vibration damper intelligent detection system.

Figure 1 presents the overall architecture of the intelligent detection system for transmission line vibration damper inspection. The system consists of three principal stages: front-end visual perception, data transmission and edge collaboration, and intelligent analysis with visualization and decision support. At the front end, images of vibration damper targets are acquired using fixed image acquisition terminals and UAV inspection terminals. The fixed terminals are employed for continuous monitoring of key line sections, whereas UAVs are used for multi-view and large-area inspection [20]. To support long-term operation in outdoor environments, the front-end nodes integrate photovoltaic power supplies, battery units, environmental monitoring devices, and temperature-control modules, thereby improving operational continuity under complex field conditions [21].

After acquisition, the images are screened, buffered, and subjected to lightweight preprocessing by edge computing nodes [22] before being transmitted to a central analysis platform through wireless transmission units or wired backhaul links [23]. The introduction of edge nodes helps reduce the transmission of invalid data, alleviate communication load, and improve the responsiveness of the overall analysis workflow. At the central platform, the proposed CARE-Net is applied to vibration damper detection and condition identification. The resulting target locations, category labels, and abnormal condition information are subsequently delivered to a visualization and warning terminal [24]. This architecture illustrates the workflow from field image acquisition and data transmission to intelligent analysis and result presentation, providing an application framework that facilitates the integration of the proposed model into a transmission line inspection platform.

**Figure 2:**
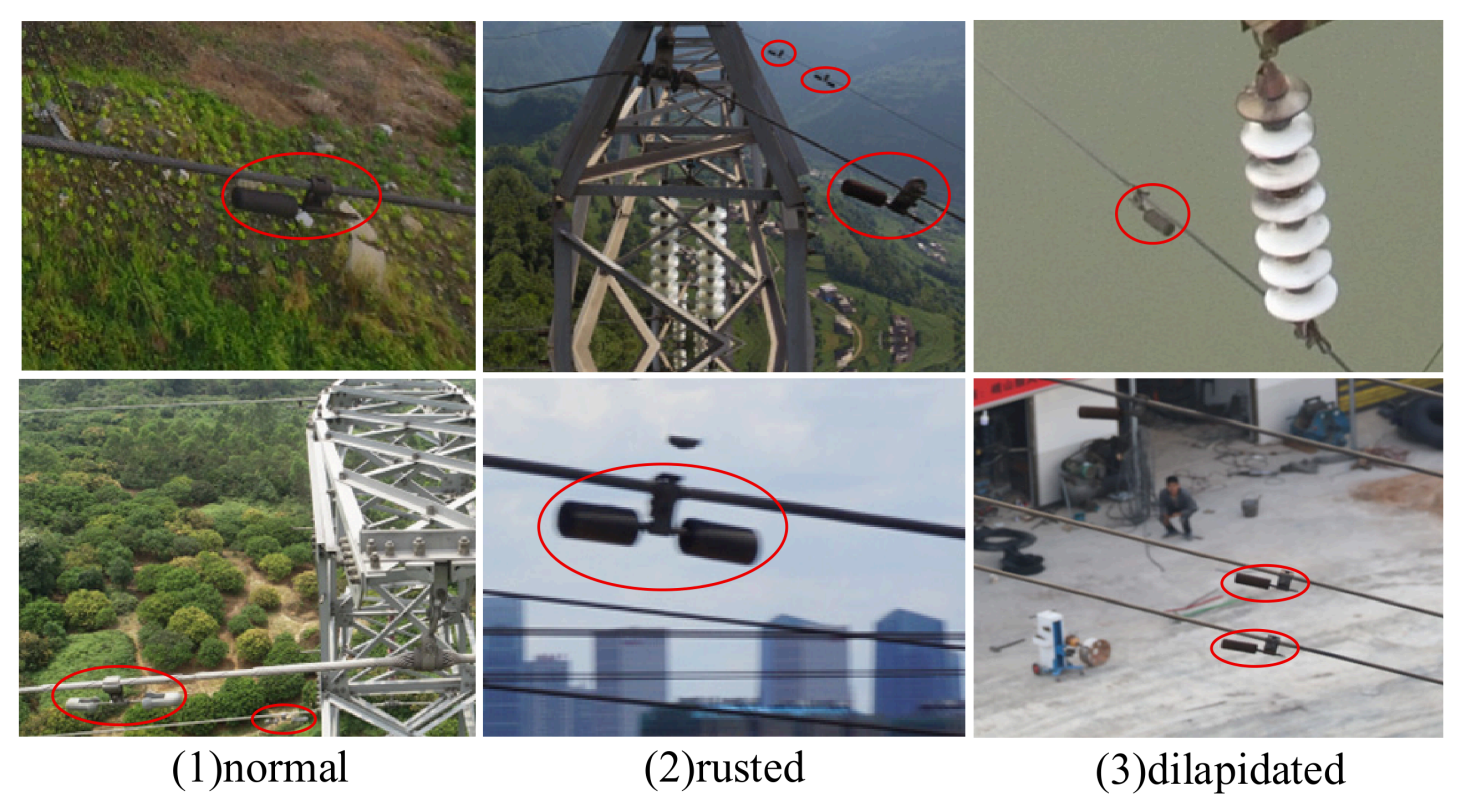
Representative UAV inspection images of vibration damper under different condition categories. The red ellipses indicate the locations of vibration damper targets for visual reference and do not represent model-generated detection results.

Figure 2 presents representative images of vibration damper targets collected in UAV-based transmission line inspection scenarios. The selected samples cover different viewing angles, spatial positions, and complex background conditions commonly encountered in practical inspection tasks. The red ellipses in the figure are used solely to indicate the locations of vibration damper targets for visual reference and do not represent detection results generated by the model. According to their observable condition characteristics, the vibration damper instances are categorized into three classes: normal, rusted, and dilapidated. These samples visually illustrate the detection challenges considered in this study, including small target scale, slender structure, and susceptibility to background interference.

#### 2.1.2. Deployment Workflow of the Inspection System

**Figure 3:**
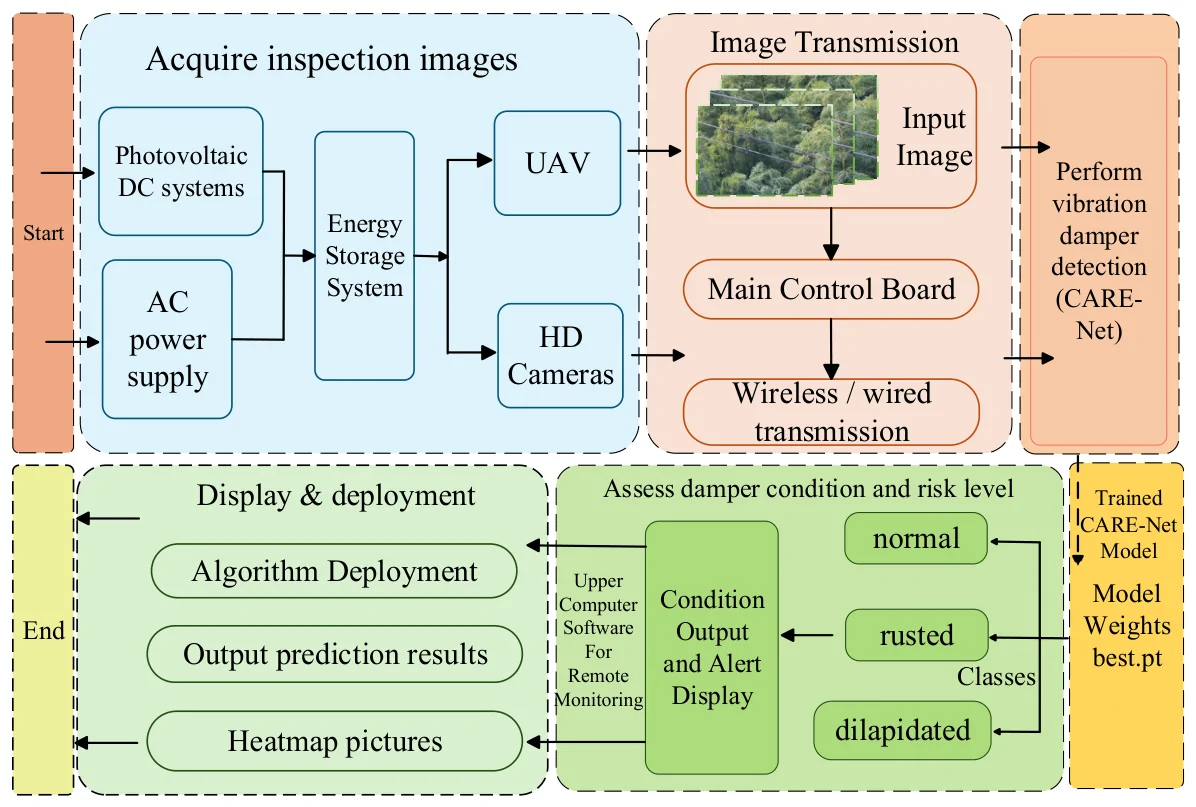
Overall workflow of the vibration damper inspection system.

Figure 3 illustrates the deployment-oriented workflow of the intelligent inspection system for vibration damper detection. At the front end, transmission line images are acquired using UAVs and high-resolution imaging devices. Photovoltaic DC power supply, AC power supply, and energy storage units are incorporated to support the sustained operation of field inspection equipment. The acquired images are coordinated by the main control unit and transmitted to the back-end computing platform through wired or wireless communication links, thereby providing inputs for subsequent intelligent analysis.

At the back-end analysis stage, the pretrained CARE-Net model is loaded to perform vibration damper detection and condition identification on the input images, producing target locations, category labels, and confidence scores. The detection outputs are subsequently delivered to a condition assessment and warning module, which generates corresponding condition judgments and abnormality alerts according to the three condition categories defined in the dataset. These results are then transmitted to the remote monitoring terminal for visualization [25]. In addition, the system is designed to support the output of detection result images and heatmaps, enabling maintenance personnel to obtain a more intuitive representation of model-responsive regions and potential fault-related evidence. By connecting image acquisition, data transmission, model inference, condition assessment, and result feedback, the workflow illustrates the potential integration of CARE-Net into a transmission line inspection analysis process and provides a basis for subsequent terminal-side software integration and operational validation.

### 2.2. CARE-Net Network Architecture

**Figure 4:**
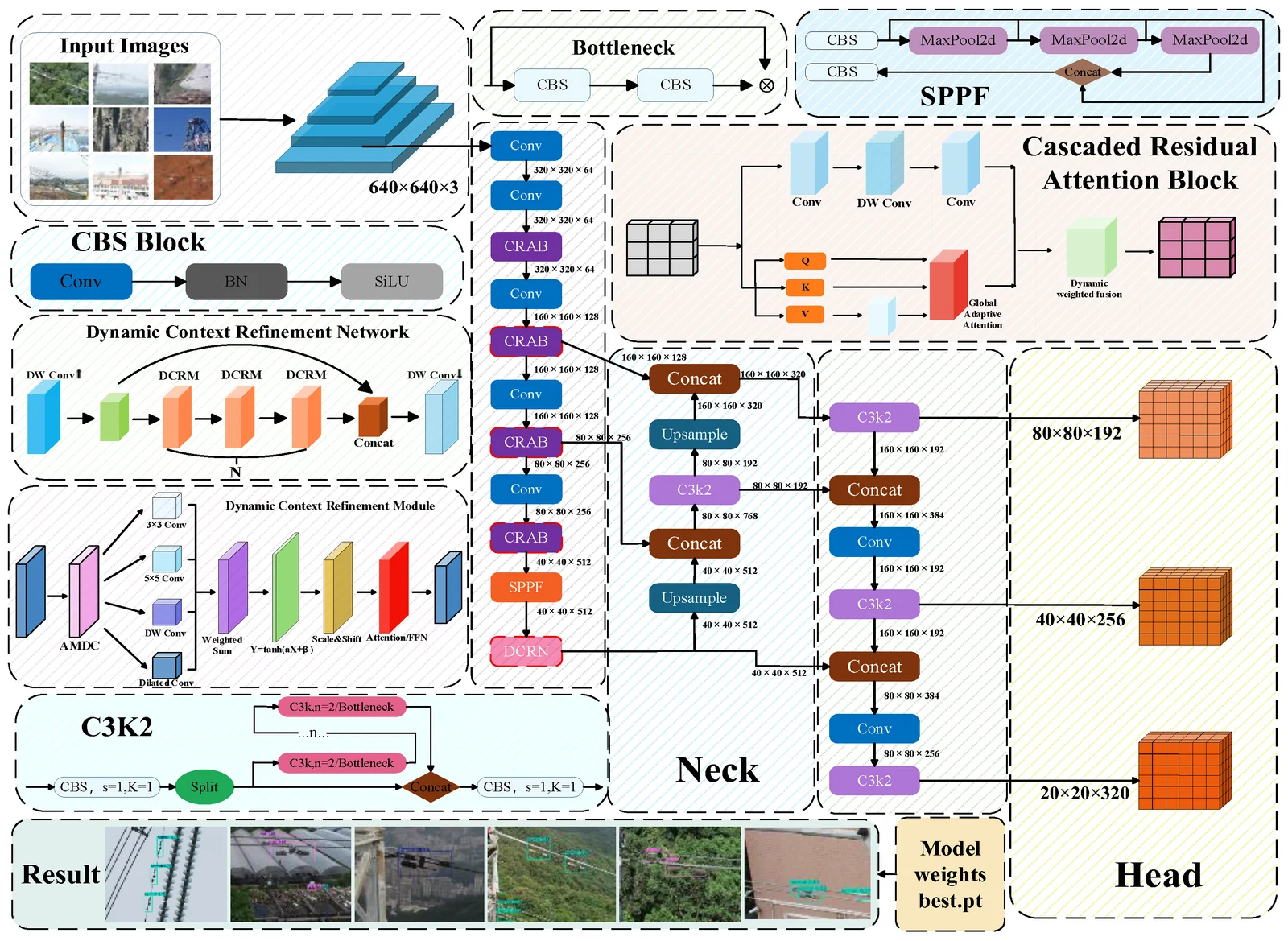
Overall architecture of the proposed CARE-Net for transmission-line vibration damper detection.

In UAV-based transmission line inspection, vibration damper targets are adversely affected by their small scale, slender structure, and interference from complex backgrounds [26]. Consequently, conventional detection networks are prone to structural detail loss and cross-scale semantic inconsistency [27]. To address these issues, this paper proposes CARE-Net, a compact framework for vibration damper detection, whose overall architecture is illustrated in Figure 4.

The proposed framework consists of three principal components: the backbone, neck, and detection head. The input image is first processed by the backbone to extract multi-scale features, which are subsequently fused across scales in the neck before the detection head generates predictions for different condition categories. CARE-Net adopts an asymmetric feature modeling strategy comprising front-end structural enhancement and back-end contextual refinement. Specifically, CRAB is deployed exclusively in the backbone to strengthen slender edge cues and local structural information of vibration damper targets, whereas DCRN is introduced at the backbone–neck transition to improve multi-scale contextual representations and semantic consistency. In addition, during training, AF-CIoU replaces the original bounding-box regression loss to further improve localization stability for slender targets through quality-aware regression constraints. This design helps mitigate potential semantic redundancy caused by repeated enhancement in high-level pyramid features and improves the detection stability of slender small objects in complex backgrounds. The red dashed boxes in Figure 4 indicate the key structural modifications introduced by CARE-Net relative to the baseline framework.

#### 2.2.1. Cascaded Residual Attention Block

**Figure 5:**
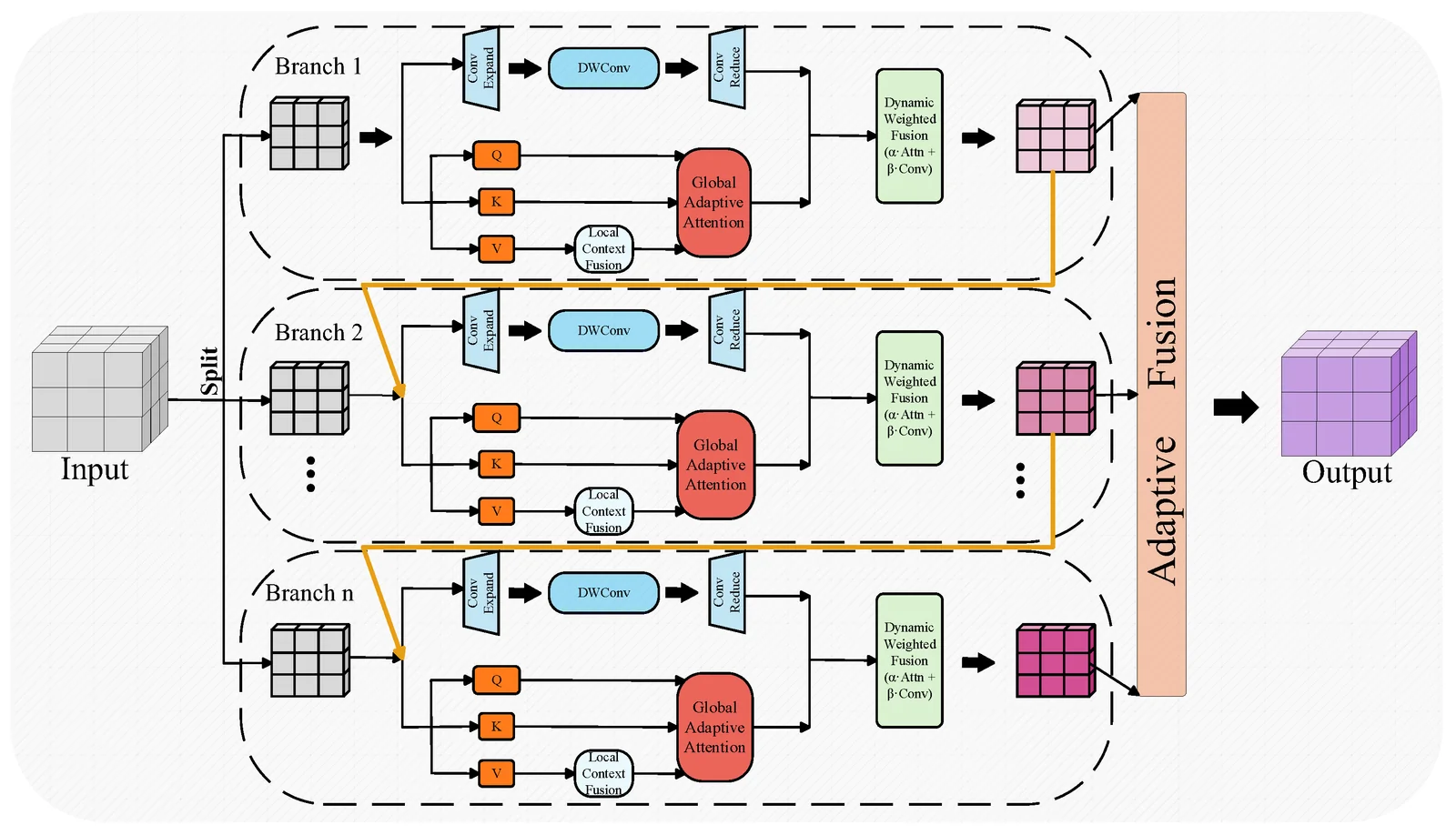
Architecture of the Cascaded Residual Attention Block. Different colors denote distinct functional components, while arrows indicate the direction of feature propagation.

To enhance the feature representation of slender structures and weak-texture regions in vibration damper targets, this paper introduces the Cascaded Residual Attention Block (CRAB), whose architecture is illustrated in Figure 5. The global modeling pathway of CRAB is inspired by efficient cascaded attention structures. Its central idea is to embed Transformer-style multi-head self-attention into a lightweight residual convolutional structure, enabling the network to jointly model local structural details and long-range semantic dependencies. Compared with feature extraction schemes relying solely on fixed convolutional receptive fields, CRAB adaptively adjusts feature responses according to the input content, making it better suited to the representation of small-scale, slender, and weak-texture vibration damper targets under long-distance imaging conditions. The effectiveness of its deployment strategy is further examined through the placement ablation study presented later in this paper.

Given an input feature map $X\in R^{C\times H\times W}$, CRAB first applies a 1 × 1 convolution for channel mapping and then partitions the resulting feature map into multiple parallel branches to capture structural responses at different scales. Each branch consists of a local convolutional refinement pathway and a global self-attention pathway. The local pathway employs a lightweight spatial modeling unit composed of a 1 × 1 convolution and a 3 × 3 depthwise convolution. Specifically, the depthwise convolution captures local edge and texture details, whereas the pointwise convolution performs inter-channel information interaction, thereby strengthening local structural representation for slender targets with limited computational overhead [28].

In the global pathway, CRAB incorporates Transformer-style multi-head self-attention to compensate for the limited capacity of local convolution in modeling long-range dependencies [29]. The input feature is linearly projected to generate the query, key, and value representations:

(1) $$Q_{i}={X_{i}W}_{Q},K_{i}={X_{i}W}_{K},V_{i}={X_{i}W}_{V}$$

Multi-head self-attention subsequently computes the correlation between *Q* and *K* to obtain global dependency weights, which are applied to *V* to generate the global contextual feature:

(2) $$A_{i}=\mathrm{MHSA}(Q_{i},K_{i},V_{i})$$

where MHSA(·) denotes the multi-head self-attention operation. Through this mechanism, CRAB establishes contextual relationships between vibration damper targets and their surrounding regions over a broader spatial range, thereby improving feature discrimination under partial occlusion, background similarity, and long-distance weak-texture imaging conditions.

The output $C_{i}$ of the local convolutional pathway is then fused with the output $A_{i}$ of the global attention pathway to obtain the feature representation of the (i)-th branch:

(3) $$Y_{i}=\alpha_{i}A_{i}+\beta_{i}C_{i}$$

where $\alpha_{i}$ and $\beta_{i}$ denote adaptive fusion weights that balance local structural details and global semantic information. The outputs of different branches are further aggregated using branch-level weighting:

(4) $$Y=\sum_{i=1}^{N}\gamma_{i}Y_{i}$$

where $\gamma_{i}$ denotes the branch-level attention weight used to adjust the contribution of structural representations at different scales. Finally, the output of CRAB is obtained through a residual connection:

(5) $$Y_{out}=Y+X$$

The residual connection preserves the fundamental structural information contained in the input feature while facilitating stable optimization in deeper networks.

#### 2.2.2. Dynamic Context Refinement Network

**Figure 6:**
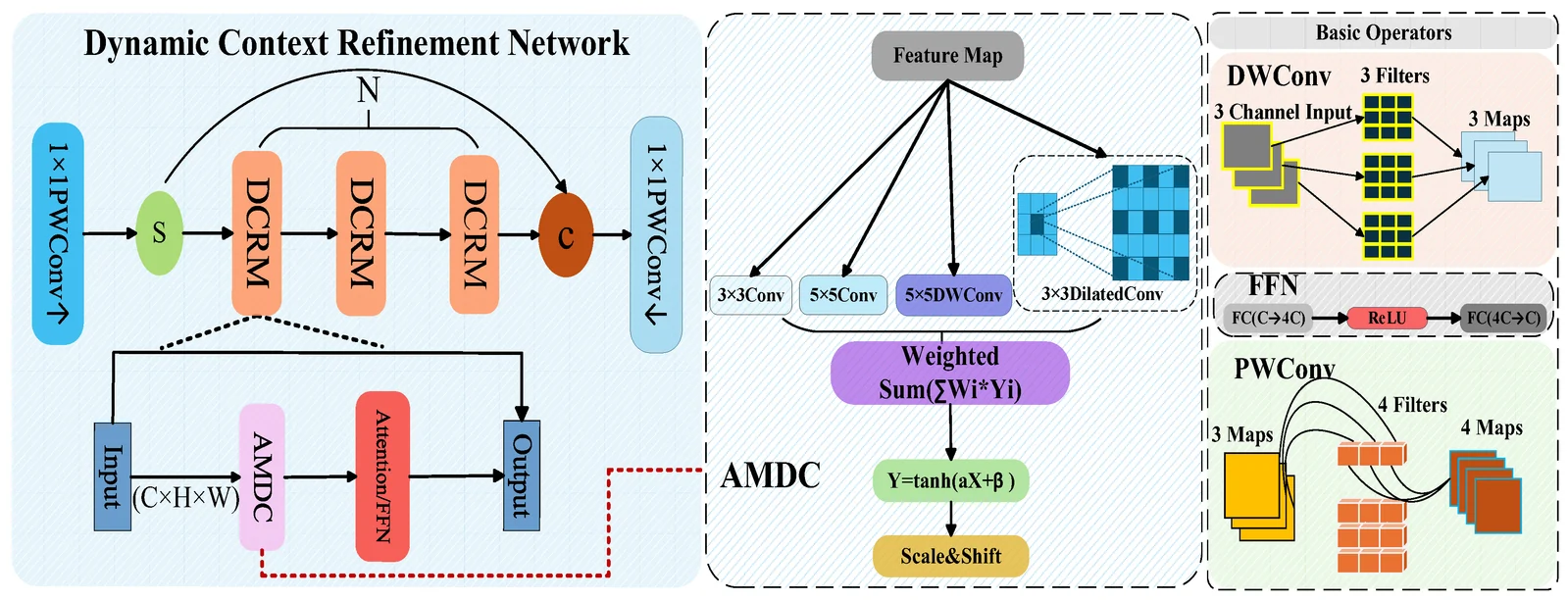
Architecture of DCRN and AMDC-based dynamic modulation. Different colors denote distinct functional components, while arrows indicate the direction of feature propagation.

To alleviate the sparse semantic responses of small-scale targets, pronounced scale variation, and insufficient multi-scale fusion in long-distance UAV inspection imagery, this paper designs a Dynamic Context Refinement Network (DCRN), whose architecture is illustrated in Figure 6. DCRN aims to perform input-adaptive contextual recalibration of deep semantic features before they enter the neck for feature fusion, thereby improving the representation stability of small-scale vibration damper targets in complex backgrounds.

As shown on the left side of Figure 6, DCRN adopts a lightweight topology similar to cross-stage partial connections. The input feature is first processed by a 1 × 1 pointwise convolution for channel mapping and is then divided into two pathways at the Split node. One part of the feature is fed into a cascaded refinement branch composed of (N) Dynamic Context Refinement Modules (DCRMs), whereas the remaining part is directly propagated to the Concat node through a skip pathway. This design preserves the original gradient information while reducing redundant computation, thereby avoiding excessive degradation of fundamental structural features during deep contextual refinement.

DCRM serves as the core refinement unit of DCRN. Within each DCRM, the input feature is first processed by Adaptive Multi-scale Dynamic Convolution (AMDC) for multi-scale dynamic modeling, and is subsequently passed through an Attention/Feed-Forward Network (FFN) branch for global contextual enhancement and nonlinear feature transformation [30]. This design enables DCRM to capture local and regional contextual information under different receptive fields, while further improving the discriminative capability of deep semantic features through attention modeling and feed-forward mapping. After multiple DCRMs are cascaded, the features are progressively refined during successive propagation, thereby improving semantic consistency among targets at different scales.

The structure of AMDC is illustrated in the middle part of Figure 6. Given an input feature *X*, AMDC employs multiple convolutional branches to extract contextual responses under different receptive fields, including a 3 × 3 convolution, a 5 × 5 convolution, a 5 × 5 depthwise convolution, and a 3 × 3 dilated convolution. The outputs of these branches are dynamically aggregated using input-dependent weights:

(6) $$Y=\sum_{i=1}^{4}w_{i}\left(X\right)Y_{i}$$

where $Y_{i}$ denotes the output feature of the (i)-th branch, and $w_{i}\left(X\right)$ represents the branch weight adaptively generated from the input feature. Through this weighted fusion process, AMDC can adaptively emphasize contextual responses associated with target scale variation, background complexity, and semantic response strength in the current image.

The fused feature is subsequently subjected to nonlinear scale-and-shift modulation:

(7) Z=s·tanh(αY+β)+t

where *α* and *β* are used to adjust the nonlinear response distribution of the fused feature, while *s* and *t* denote the scaling and shifting parameters in the Scale & Shift operation, respectively. This process corresponds to the “Weighted Sum → tanh(*α*X + *β*) → Scale & Shift” flow shown in Figure 6. It further enhances the adaptability of the feature representation to variations in the input distribution, thereby alleviating the inflexibility of fixed convolutional responses in complex inspection scenarios.

Finally, the output of the cascaded DCRM branch is fused with the feature propagated through the skip pathway at the Concat node, followed by a 1 × 1 pointwise convolution for channel integration to obtain the output feature of DCRN. Overall, DCRN employs a lightweight “Split–DCRM–Concat” topology to dynamically refine deep semantic features while preserving fundamental structural information. AMDC further strengthens contextual adaptability through multi-scale dynamic convolution and nonlinear modulation. This design provides more stable and semantically consistent high-quality feature representations that are responsive to scale variation for subsequent feature fusion in the neck.

#### 2.2.3. Adaptive Focal Complete IoU Loss

CARE-Net is constructed based on the YOLOv11 detection framework. Considering the small scale, slender contours, and boundary-sensitive localization characteristics of vibration damper targets, this paper adopts a focal-weighted Complete IoU regression strategy, referred to as AF-CIoU in the CARE-Net framework. The overall detection loss is formulated as:

(8) $$L_{det}=\lambda_{cls}L_{cls}+\lambda_{box}L_{AF-CIoU}+\lambda_{dfl}L_{DFL}$$

where $L_{cls}$ denotes the classification loss, $L_{AF-CIoU}$ represents the bounding-box regression loss used in CARE-Net, and $L_{DFL}$ denotes the Distribution Focal Loss used for bounding-box distribution modeling. The coefficients $\lambda_{cls}$, $\lambda_{box}$, $\lambda_{dfl}$ are the corresponding weighting factors. Since AF-CIoU is applied only to bounding-box regression during training, it introduces neither additional prediction branches nor extra parameters or computational complexity during inference.

Complete IoU (CIoU) loss [31] improves the geometric completeness of bounding-box regression by jointly constraining the overlap, center-point distance, and aspect-ratio consistency between the predicted and ground-truth boxes. It is defined as:

(9) $$L_{CIoU}=1-IoU+\frac{\rho^{2}(b,b^{\mathrm{gt}})}{c^{2}}+\alpha \nu$$

where IoU denotes the intersection over union between the predicted box B and the ground-truth box $B^{gt}$; b and $b^{gt}$ represent their center coordinates, respectively; *ρ*(·) denotes the Euclidean distance between the two center points; c is the diagonal length of the smallest enclosing box covering both the predicted and ground-truth boxes; and v represents the aspect-ratio consistency term, defined as:

(10) $$\nu =\frac{4}{\pi^{2}}(\arctan\frac{w^{gt}}{h^{gt}}-(\arctan\frac{w}{h}{)}^{2})$$

(11) $$\alpha =\frac{\nu }{1-\mathrm{IoU}+\nu +\varepsilon }$$

where w and h denote the width and height of the predicted bounding box, respectively; $w^{gt}$ and $h^{gt}$ denote those of the ground-truth bounding box; and *ε* is a small constant introduced to ensure numerical stability.

Although CIoU simultaneously constrains the overlap ratio, center-point displacement, and aspect-ratio discrepancy, it does not explicitly differentiate the regression contributions of samples with different localization qualities. Inspired by existing focal reweighting strategies [32], an IoU-dependent weighting mechanism is introduced to adaptively adjust the regression contribution according to localization quality. For small and slender vibration damper targets that are susceptible to localization deviations in complex inspection scenes, the adaptive focal weighting term is formulated on the basis of CIoU as follows:

(12) $$w_{foc}=(1-\tilde{\mathrm{IoU}}{)}^{\gamma },\tilde{\mathrm{IoU}}=\mathrm{clip}(\mathrm{IoU},0,1)$$

where γ > 0 is the focal modulation parameter used to control the weight differences among samples with different localization qualities. In this study, γ is set to 1.5 to provide a moderate focal modulation strength, balancing the emphasis on difficult regression samples and the suppression of well-localized samples. It should be noted that the adaptiveness of AF-CIoU originates from the dynamic response of the regression weight to the matching quality of each predicted sample, rather than from an additional learnable prediction branch. Accordingly, the regression contribution of each sample can be adjusted according to its localization quality without modifying the detection architecture. AF-CIoU is defined as follows:

(13) $$L_{AF-CIoU}=w_{foc}L_{CIoU}=(1-\tilde{\mathrm{IoU}}{)}^{\gamma }[1-IoU+\frac{\rho^{2}(b,b^{\mathrm{gt}})}{c^{2}}+\alpha \nu ]$$

In implementation, the IoU-dependent focal weighting term is computed directly from the predicted-box IoU without applying detach or stop-gradient operations; therefore, it remains in the computational graph and participates in gradient propagation during backpropagation. As indicated by Equation (13), AF-CIoU preserves the complete geometric constraints of CIoU while dynamically adjusting the regression contribution according to the matching quality between the predicted and ground-truth bounding boxes. For predictions with relatively high localization quality, the focal weight decreases as the IoU increases, thereby reducing repeated optimization of well-localized samples. For predictions with relatively low matching quality, their relative regression contributions are retained, which helps improve the localization of challenging targets under complex backgrounds and long-distance imaging conditions.

**Figure 7:**
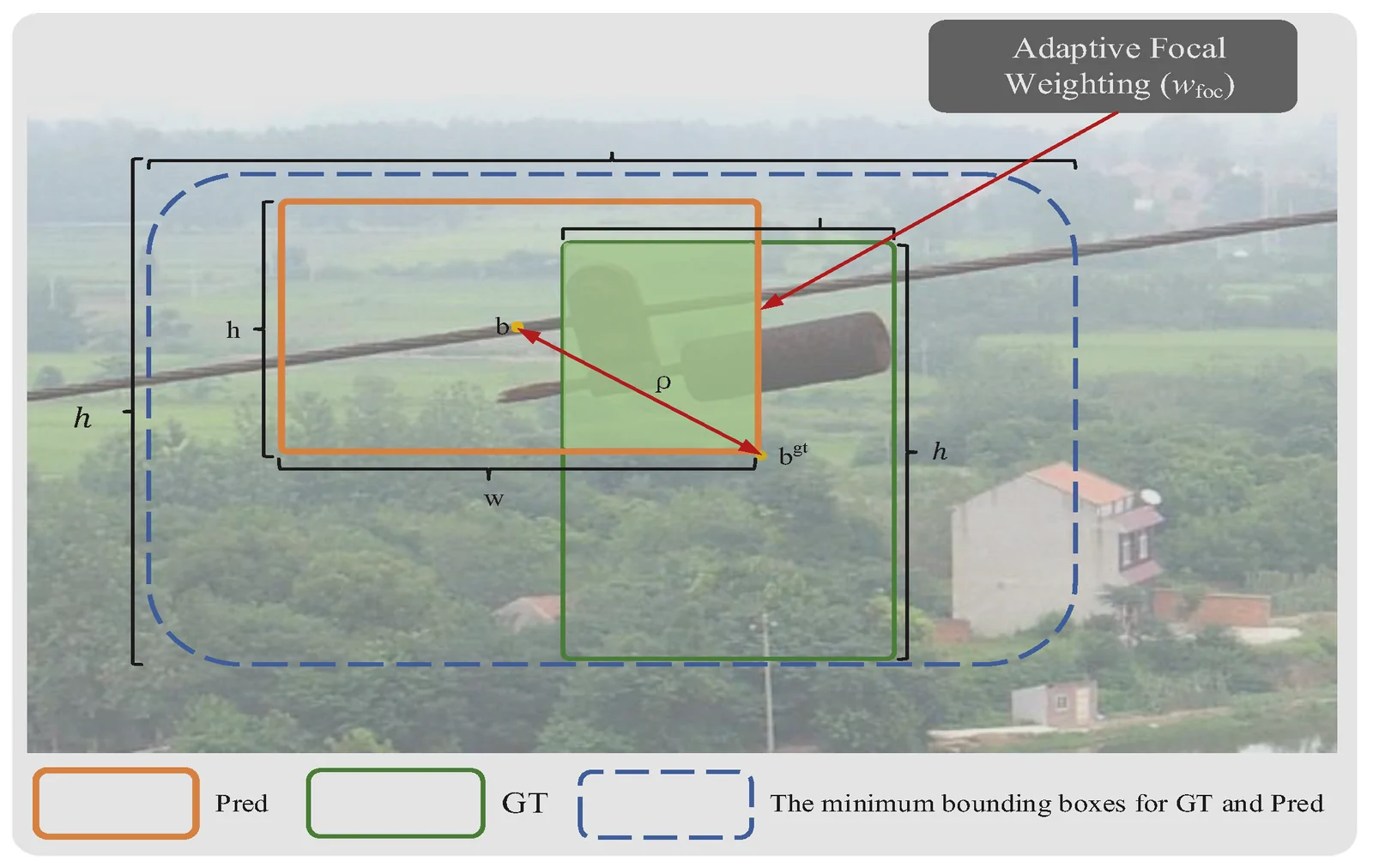
Geometric interpretation of the proposed AF-CIoU with adaptive focal weighting.

Figure 7 illustrates the geometric interpretation of AF-CIoU. This loss jointly considers the overlap relationship, center-point displacement, and shape discrepancy between the predicted and ground-truth bounding boxes, while optimizing the contribution allocation of regression samples through an adaptive focal weighting mechanism. Since AF-CIoU is applied only to loss calculation during training, it does not alter the inference architecture of CARE-Net or introduce additional parameters and computational complexity during inference. Therefore, it helps improve target localization quality while maintaining model compactness. A quantitative comparison of different bounding-box regression losses is further provided in Section 3.3.5.

## 3. Experiments and Results

### 3.1. Datasets Construction and Data Acquisition

The vibration damper dataset used in this study was collected from practical UAV-based transmission line inspection tasks. The acquired images cover different flight viewpoints, imaging distances, illumination conditions, and complex background scenes to closely approximate practical engineering inspection environments. Unlike publicly available general-purpose object detection datasets, this dataset focuses on the specific small-object scenario of vibration damper detection in transmission lines. The samples include fully visible targets, partially occluded targets, long-distance imaging cases, weak-texture targets, and background interference from conductors, towers, and vegetation. Therefore, the dataset reflects common challenges in practical inspection, including scale variation and visual similarity between targets and backgrounds.

The dataset consists of 1477 images, all of which were manually annotated using LabelImg (HumanSignal, available at https://github.com/HumanSignal/labelImg, accessed on 23 July 2026). Each vibration damper instance was assigned a bounding box and its corresponding class label. According to typical conditions observed in practical inspections, vibration damper targets were categorized into three classes: normal, rusted, and dilapidated, thereby supporting unified detection and recognition of targets in normal and abnormal conditions. To ensure consistency in condition labeling, annotation criteria were established according to visible appearance characteristics. Specifically, normal indicates that the structure is intact, with no evident corrosion or damage observed; rusted indicates that the structure remains intact but contains clearly visible corroded regions on its surface; and dilapidated indicates abnormalities such as missing hammer heads, damaged connecting structures, or evident structural incompleteness. For targets simultaneously exhibiting corrosion and structural damage, the dilapidated label was assigned preferentially. All annotations were cross-checked by two annotators, and samples with inconsistent judgments were further reviewed.

During dataset construction, no additional offline data augmentation strategy was introduced to preserve the original distribution of the inspection images. The 1477 images used for dataset partitioning were independent original samples rather than adjacent frames extracted from continuous UAV video sequences, and no augmented copies derived from the same original image were distributed across different subsets. After annotation, the complete dataset was randomly divided into training, validation, and test sets at a ratio of 7:2:1. The training set was used for model parameter learning, the validation set was used for model selection, module ablation, loss-function comparison, hyperparameter adjustment, and convergence monitoring, whereas the test set was reserved exclusively for final independent performance evaluation and did not participate in model architecture or hyperparameter selection. The same data partition was adopted for all comparative models to ensure fairness and reproducibility. However, route-level, acquisition-session-level, conductor-span-level, and physical-instance-level identifiers were not retained during dataset construction; therefore, the current split should be interpreted as an image-level evaluation rather than a strict cross-route or cross-instance generalization test. In addition, the relatively limited size of the current independent test set restricts a more comprehensive statistical assessment of generalization performance, which will be addressed through larger and more diverse inspection datasets in future work.

### 3.2. Experimental Settings and Evaluation Metrics

To ensure the fairness and reproducibility of the experimental results, all models were trained and tested under a unified hardware and software environment. The experiments were conducted on an Ubuntu 18.04 workstation equipped with an Intel i9-11900K CPU, 64 GB of RAM, and an NVIDIA RTX 3090 GPU. All methods were implemented using the PyTorch framework, with Python 3.9.0, PyTorch 1.8.0, and CUDA 11.1 used in the experimental environment.

During training, all models adopted the same dataset split and maximum number of training epochs. The maximum number of epochs was set to 300, with a batch size of 16. Stochastic gradient descent (SGD) was employed as the optimizer, with an initial learning rate of 0.001, a momentum of 0.9, and a weight decay of 0.0005. For the YOLO-series models and the proposed method, the input image size was set to 640 × 640. For Faster R-CNN, SSD, CenterNet, EfficientDet, and RetinaNet, training and testing were conducted using their commonly adopted or officially recommended input sizes, allowing each model to participate in the comparison under a reasonable configuration. Precision, Recall, mAP^@0.5^, mAP^@0.5:0.95^, Params, and GFLOPs were adopted as the primary evaluation metrics. Specifically, Precision and Recall measure detection accuracy and target retrieval capability, respectively; mAP^@0.5^ reflects the overall detection performance; mAP^@0.5:0.95^ evaluates detection performance over IoU thresholds ranging from 0.5 to 0.95 and provides a stricter assessment of localization quality; Params and GFLOPs characterize the model size and computational complexity, respectively.

Precision measures the proportion of correctly predicted positive samples among all predicted positives and is defined as:

(14) $$Precision=\frac{TP}{TP+FP}$$

where TP and FP denote true positives and false positives, respectively.

Recall reflects the proportion of correctly detected positive samples among all ground-truth positives and is given by:

(15) $$Recall=\frac{TP}{TP+FN}$$

where FN denotes false negatives.

The Average Precision (AP) is calculated as the area under the precision–recall (P–R) curve:

(16) $$AP=\int_{0}^{1}P(r)dr$$

The mean Average Precision (mAP) is obtained by averaging the AP values across all object categories:

(17) $$mAP=\frac{1}{N}\sum_{i=1}^{N}AP_{i}$$

### 3.3. Result Analysis

#### 3.3.1. Ablation Experiment

To evaluate the effectiveness of each key component in CARE-Net, all ablation and configuration-selection experiments were evaluated on the validation set under identical training configurations and dataset splits. The component ablation results are presented in Table 1. In the following tables, bold values indicate the best performance for each metric, while “√” denotes that the corresponding module is included in the model configuration.

The results obtained with individual components indicate that each component contributes to a different aspect of model optimization. After replacing the corresponding original block with CRAB, the number of parameters decreases from 2.58 M to 2.44 M and the computational complexity decreases from 6.3 GFLOPs to 6.2 GFLOPs, while a slight improvement is achieved in mAP^@0.5:0.95^. This result indicates that deploying CRAB in the backbone provides useful representational support for the slender contours and local structures of vibration damper targets while maintaining a compact model structure. When DCRN is introduced independently, Recall increases from 0.889 to 0.911, indicating that dynamic contextual refinement is more effective in preserving target response integrity under complex backgrounds and reducing the risk of missed detections. However, the two mAP metrics do not improve correspondingly, suggesting that contextual modeling alone is insufficient to consistently address the precise localization of slender targets. Notably, when AF-CIoU is individually applied to the baseline model, Precision increases, whereas Recall and both mAP metrics show no improvement. This observation indicates that, for vibration damper targets characterized by slender structures, weak textures, and susceptibility to background interference, regression loss optimization cannot function independently of feature representation quality. When the features provided to the regression branch do not sufficiently preserve target structural cues or establish stable contextual responses, quality-aware weighting for challenging samples cannot be fully translated into overall localization gains.

When CRAB and DCRN are jointly introduced, the model achieves an mAP^@0.5^ of 0.941 and an mAP^@0.5:0.95^ of 0.619, both exceeding those of the baseline model, while the parameter count and computational complexity remain at 2.44 M and 6.2 GFLOPs, respectively. This result demonstrates the complementarity between front-end structural enhancement and back-end contextual refinement. Through their combined effect, the model obtains a more suitable feature basis for subsequent classification and localization optimization. Furthermore, after AF-CIoU is incorporated on this basis, the complete CARE-Net achieves the best overall detection results, with Precision, Recall, mAP^@0.5^, and mAP^@0.5:0.95^ reaching 0.967, 0.922, 0.951, and 0.628, respectively. Compared with the baseline model, both mAP metrics increase by 0.013. The difference between the AF-CIoU-only configuration and the complete configuration further indicates that quality-aware bounding-box regression optimization is more effective when built upon stable structural representation and contextual expression. Once CRAB and DCRN have improved the quality of target features, AF-CIoU can further act on predictions with localization difficulty, thereby improving the overall regression quality of slender vibration damper targets.

Overall, the ablation results reveal the functional compatibility among the three design components of CARE-Net. For vibration damper targets in complex inspection backgrounds, the preservation of front-end structural cues provides the basis for target discrimination, deep contextual refinement further improves the integrity of target responses, and regression loss optimization subsequently enhances localization quality. While achieving the best detection performance, the complete CARE-Net retains fewer parameters and lower computational complexity than the baseline model. This result indicates that the proposed design improves the detection accuracy and localization stability of vibration damper targets while maintaining a compact model scale. To further assess the robustness of the ablation conclusions, the principal configurations were independently evaluated using three different random seeds under identical data partitions and training settings. The repeated-run results showed an overall trend consistent with the single-run ablation results, while the complete CARE-Net maintained the best overall performance, indicating that the reported improvement is not solely attributable to training randomness.

#### 3.3.2. Feature Visualization Analysis of Ablation Modules

**Figure 8:**
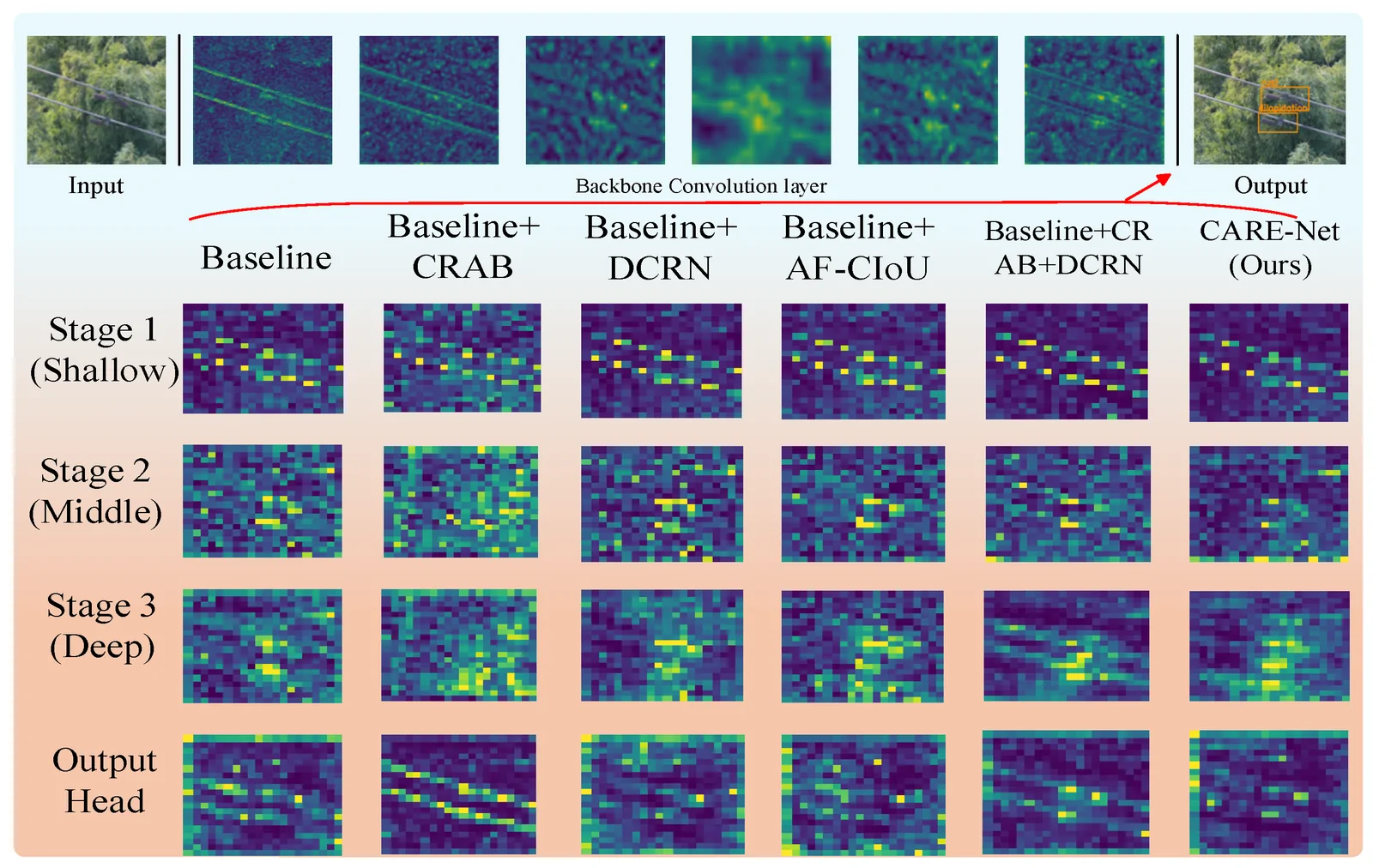
Visualization of multistage feature maps under different module configurations.

To qualitatively evaluate the effects of different components on feature representation, the shallow-, intermediate-, and deep-level feature responses under different configurations are visualized in Figure 8. As shown in the comparison, the baseline model produces relatively dispersed feature responses, with limited discrimination between vibration damper target regions and complex backgrounds. When only CRAB is introduced, local responses associated with the edges and slender structures of vibration damper targets are enhanced. By contrast, the independent introduction of DCRN leads to more concentrated intermediate- and high-level feature distributions, indicating that dynamic contextual refinement helps improve multi-scale semantic consistency. When CRAB and DCRN are jointly incorporated, the activations within the target regions become more continuous, while responses induced by background interference are suppressed. The complete CARE-Net further exhibits more compact and stable target responses at the output stage. Since AF-CIoU functions as a regression constraint during training, it does not directly alter the forward feature extraction process; instead, it implicitly promotes the aggregation of localization-relevant features through the optimization of parameter updates.

These observations indicate that the performance improvement of CARE-Net is not solely reflected in the final detection-head outputs, but is also manifested in the progressive reinforcement of target structural information during feature propagation. The visualization results in Figure 8 are consistent with the ablation results reported in Table 1, providing visual evidence that complements the quantitative analysis of the contributions of the different components.

#### 3.3.3. Comparative Analysis of CRAB Module Placement

**Figure 9:**
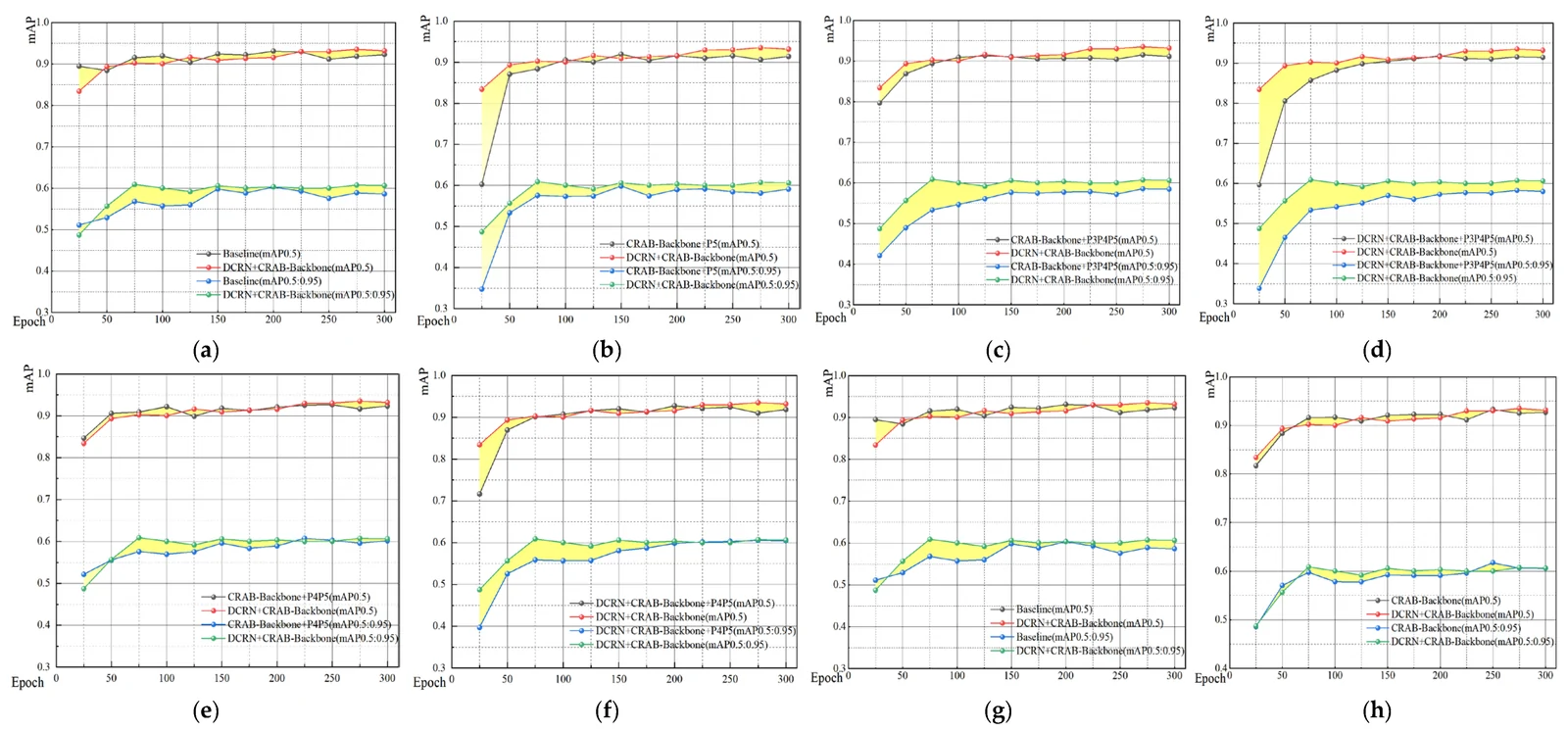
Comparison of detection performance and optimization stability under different module configurations of CRAB and DCRN: (**a**) Baseline; (**b**) CRAB in Backbone; (**c**) CRAB in Backbone and Neck P3P4P5; (**d**) CRAB in Backbone and Neck P3P4P5 with DCRN; (**e**) CRAB in Backbone and Neck P4P5; (**f**) CRAB in Backbone and Neck P4P5 with DCRN; (**g**) CRAB in Backbone and Neck P5; (**h**) CRAB in Backbone and Neck P5 with DCRN.

**Figure 10:**
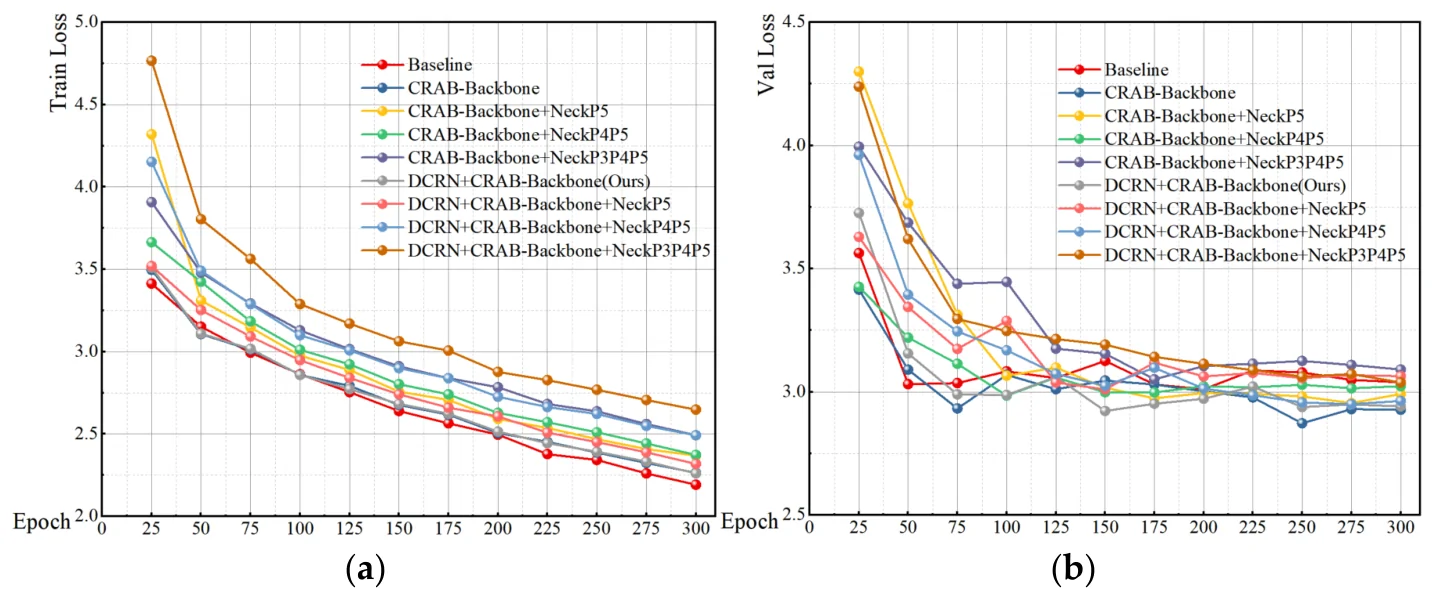
Training and validation loss function curves of different CRAB positions. (**a**) Total training loss; (**b**)Total validation loss.

Since CRAB integrates local convolutional modeling with global attention enhancement, its deployment position directly affects feature representation and optimization stability. In the vibration damper detection task, shallow-level features mainly preserve local information, such as edges, textures, and slender structural cues, whereas deep-level features are more oriented toward semantic abstraction and contextual associations. Therefore, features at different levels may respond differently to attention enhancement. To determine an appropriate deployment position for CRAB, ablation experiments were conducted by introducing CRAB into the backbone and into different feature pyramid levels of the neck, including P3, P4, and P5. The corresponding results are presented in Table 2, Figure 9, and Figure 10.

As shown in Table 2, deploying CRAB only in the backbone yields favorable results, with mAP^@0.5^ and mAP^@0.5:0.95^ reaching 0.937 and 0.617, respectively. When DCRN is further incorporated on this basis, mAP^@0.5^ increases to 0.941, while Precision and Recall reach 0.949 and 0.899, respectively. These results indicate that backbone-level CRAB deployment provides a more stable structural feature basis for subsequent dynamic contextual refinement, whereas DCRN further enhances the contextual representation capability of the target regions. In contrast, extending CRAB to high-level pyramid features in the neck does not yield consistent gains. The mAP^@0.5^ values of the Backbone + P5 and Backbone + P4 + P5 configurations decrease to 0.931 and 0.923, respectively. More notably, simultaneously stacking CRAB across the backbone and all P3, P4, and P5 levels causes mAP^@0.5^ and mAP^@0.5:0.95^ to decrease sharply to 0.402 and 0.192, respectively. This phenomenon indicates that, under the vibration damper detection task and the current module configuration considered in this study, indiscriminately extending CRAB to high-level pyramid features may introduce redundant responses and interfere with cross-scale feature fusion, thereby weakening the optimization stability and detection performance of the model.

Figure 9 and Figure 10 further support this conclusion from the perspective of training dynamics. The backbone-level CRAB deployment strategy exhibits more stable loss variation, whereas configurations that extensively extend CRAB into the neck are more susceptible to performance fluctuations and reduced optimization stability during validation. Overall, CRAB is more appropriately deployed in the backbone for front-end structural enhancement, whereas DCRN is employed for back-end contextual refinement. Together, they constitute an asymmetric design of front-end structural enhancement and back-end contextual refinement, which helps alleviate redundant responses in high-level semantic features while maintaining a favorable balance among detection accuracy, training stability, and computational efficiency. This configuration therefore provides an appropriate architectural basis for compact vibration damper detection in transmission line inspection.

#### 3.3.4. Comparison with Representative Alternative Configurations

To further verify the effectiveness of CRAB and DCRN, representative existing lightweight attention and dynamic convolution methods were introduced for controlled comparison under the same deployment positions and training settings. Specifically, ECA and CBAM were integrated into the original C3k2 structure for comparison with CRAB, while ODConv was integrated into the original C2PSA structure for comparison with DCRN. The input/output dimensions, network architecture, dataset partition, and training settings were kept unchanged. The corresponding results are presented in Table 3.

As shown in Table 3, although C3k2+ECA and C3k2+CBAM achieve slightly higher mAP^@0.5^ values than CRAB, CRAB obtains the highest mAP^@0.5:0.95^ of 0.617 while reducing the parameter count to 2.44 M and the computational complexity to 6.2 GFLOPs. In the dynamic modeling comparison, DCRN achieves mAP^@0.5^ and mAP^@0.5:0.95^ values of 0.933 and 0.616, respectively, outperforming C2PSA+ODConv under comparable computational complexity. These results provide additional evidence for the effectiveness and suitability of CRAB and DCRN in compact vibration damper detection.

#### 3.3.5. Comparison of Bounding-Box Regression Losses

After determining the key component configuration and the deployment position of CRAB, the compatibility between AF-CIoU and the feature representation framework of CARE-Net was further evaluated. With the optimal structural configuration of CRAB and DCRN fixed, comparative experiments were conducted by replacing only the bounding-box regression loss function. CIoU, EIoU, GIoU, ShapeIoU, SIoU, and DIoU were selected as alternative regression losses. All experiments were performed using the same dataset split and training settings, and the results are presented in Table 4.

As shown in Table 4, the evaluated regression losses produce different localization performance in vibration damper detection. The proposed AF-CIoU achieves the best results in Recall, mAP^@0.5^ and mAP^@0.5:0.95^, while maintaining a high Precision value second only to that of DIoU. These results indicate that, based on the stable structural representation and contextual information provided by CRAB and DCRN, AF-CIoU can further improve the bounding-box localization quality of slender vibration damper targets. Therefore, AF-CIoU is adopted as the bounding-box regression loss of CARE-Net in this study.

### 3.4. Comparative Experiment

To evaluate the overall performance of CARE-Net in the transmission line vibration damper detection task, several representative object detection algorithms were selected for comparison, including YOLOv8, YOLOv11, YOLOv12 [33], Mamba-YOLO [34], SSD [35], Faster R-CNN [36], CenterNet [37], EfficientDet [38], RetinaNet [39], Hyper-YOLO-T [40], and RT-DETR-R18 [41]. All models were trained and tested using the same dataset split and evaluation protocol, with their input sizes configured as listed in Table 5.

Considering that the proposed method is designed for compact vibration damper detection in UAV-based inspection scenarios, the comparison focuses on model size, computational complexity, detection accuracy, and localization quality. As shown in Table 5, CARE-Net achieves the best overall detection accuracy while maintaining a compact model scale. It contains 2.44 M parameters, the lowest among all compared models, and requires 6.2 GFLOPs, which is higher only than that of EfficientDet. Meanwhile, CARE-Net achieves an mAP^@0.5^ of 0.951, and an mAP^@0.5:0.95^ of 0.628. Its Precision reaches 0.967, second only to CenterNet, while its Recall remains at a high level of 0.922. These results indicate that the advantage of CARE-Net lies not in maximizing a single metric, but in achieving a favorable balance among detection accuracy, localization quality, and model complexity for vibration damper detection.

Compared with the baseline YOLOv11, CARE-Net reduces the parameter count from 2.58 M to 2.44 M and the computational complexity from 6.3 GFLOPs to 6.2 GFLOPs. Despite the reduced model size and computational complexity, Precision, Recall, and mAP^@0.5^ increase from 0.927, 0.889, and 0.938 to 0.967, 0.922, and 0.951, respectively. Meanwhile, mAP^@0.5:0.95^ increases from 0.615 to 0.628, further indicating improved localization quality under stricter IoU thresholds. In particular, the improvement in Recall indicates that the proposed method improves the completeness of vibration damper target detection in complex inspection images. For transmission line condition recognition tasks, reducing missed detections contributes to improving the reliability of subsequent abnormal-condition assessment and maintenance decision-making.

Compared with more recent one-stage detection methods, such as YOLOv12 and Mamba-YOLO, CARE-Net requires substantially fewer parameters and lower computational complexity. YOLOv12 and Mamba-YOLO contain nearly 6 M parameters and both require 13.6 GFLOPs, whereas their mAP^@0.5^ values are 0.930 and 0.928, respectively, lower than the 0.951 achieved by CARE-Net. Hyper-YOLO-T achieves an mAP^@0.5^ of 0.943 and an mAP^@0.5:0.95^ of 0.610 with 2.68 M parameters and 7.7 GFLOPs, whereas RT-DETR-R18 obtains 0.931 and 0.607 for the two mAP metrics, respectively, with 21.32 M parameters and 60.5 GFLOPs. In comparison, CARE-Net achieves 0.951 and 0.628 in mAP^@0.5^ and mAP^@0.5:0.95^, respectively, while maintaining lower model complexity. These results indicate that, for vibration damper targets characterized by slender structures and weak textures, relying solely on a more complex general-purpose detection architecture does not necessarily result in better task adaptability. Instead, targeted optimization of structural cue preservation, deep contextual organization, and bounding-box localization quality is more consistent with the detection requirements of such targets.

The comparison among different detection paradigms further supports this observation. CenterNet achieves the highest Precision, but its parameter count and computational complexity reach 32.67 M and 35.1 GFLOPs, respectively. RetinaNet and Faster R-CNN obtain relatively high Recall values; however, their computational complexity reaches 55.3 GFLOPs and 470.5 GFLOPs, respectively, and their mAP^@0.5^ values remain lower than that of CARE-Net. By contrast, although EfficientDet achieves the lowest computational complexity, its Recall and mAP^@0.5^ are markedly lower than those of CARE-Net. These results suggest that model design for vibration damper inspection tasks should jointly consider target detection completeness, overall detection accuracy, and computational burden, rather than evaluating its application value on the basis of a single metric alone.

**Figure 11:**
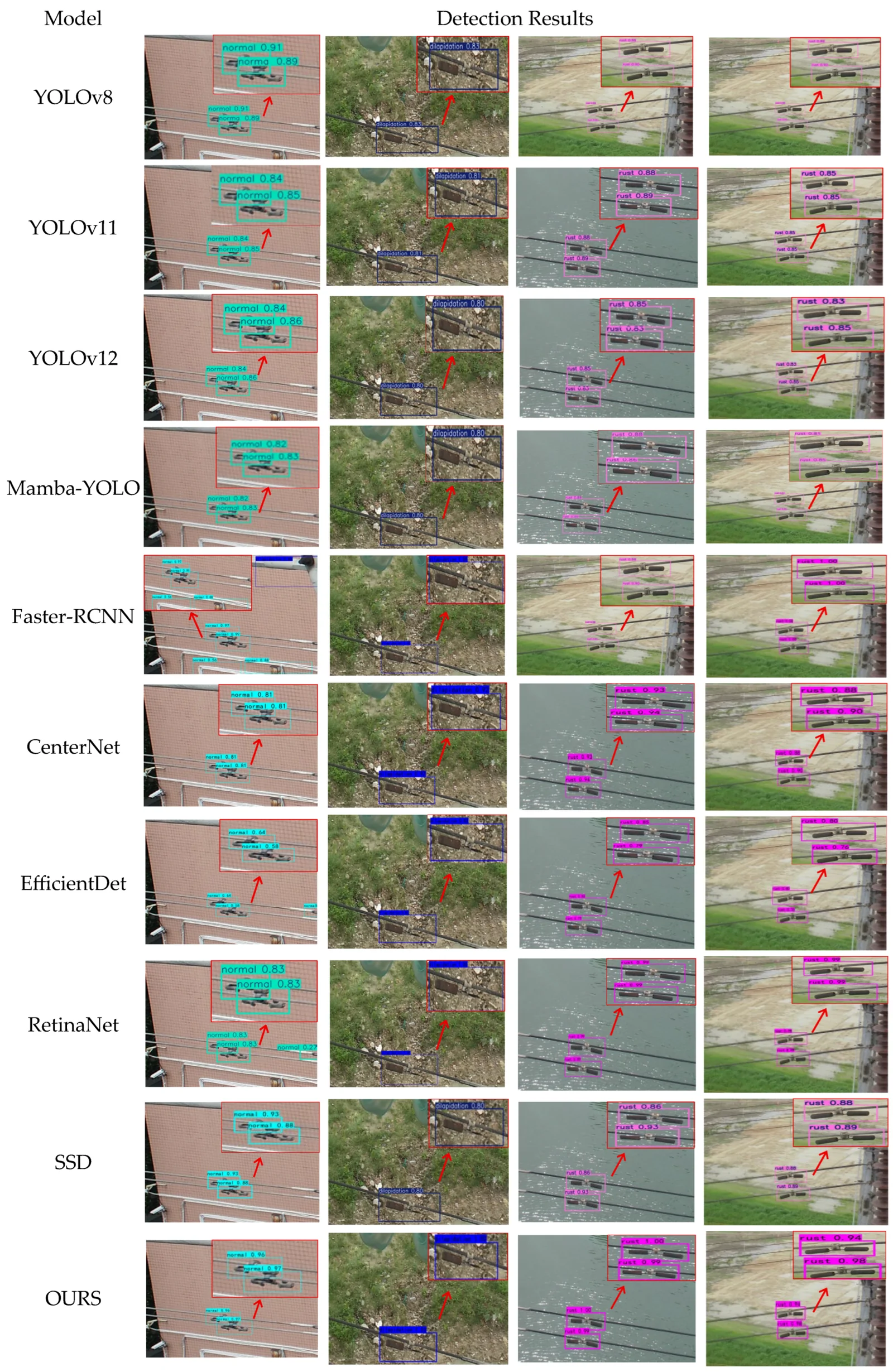
Comparison of visualization results. Different colors denote different condition categories, while the red arrows indicate regions selected for enlarged visualization.

Overall, CARE-Net achieves the highest mAP^@0.5^ and mAP^@0.5:0.95^ with the lowest parameter count and relatively low computational complexity, while maintaining competitive Precision and Recall. These results support the effectiveness of the staged optimization strategy adopted in this study: front-end structural enhancement helps preserve discriminative cues for slender targets, back-end contextual refinement improves feature organization under complex backgrounds, and quality-aware regression optimization complements the overall detection performance. Accordingly, CARE-Net provides a compact detection approach with favorable overall performance for intelligent analysis of vibration damper conditions in transmission line inspection. To further provide an intuitive comparison of detection performance under representative inspection scenarios, Figure 11 presents qualitative results for several representative detectors covering different architectural paradigms rather than all methods listed in Table 5.

**Figure 12:**
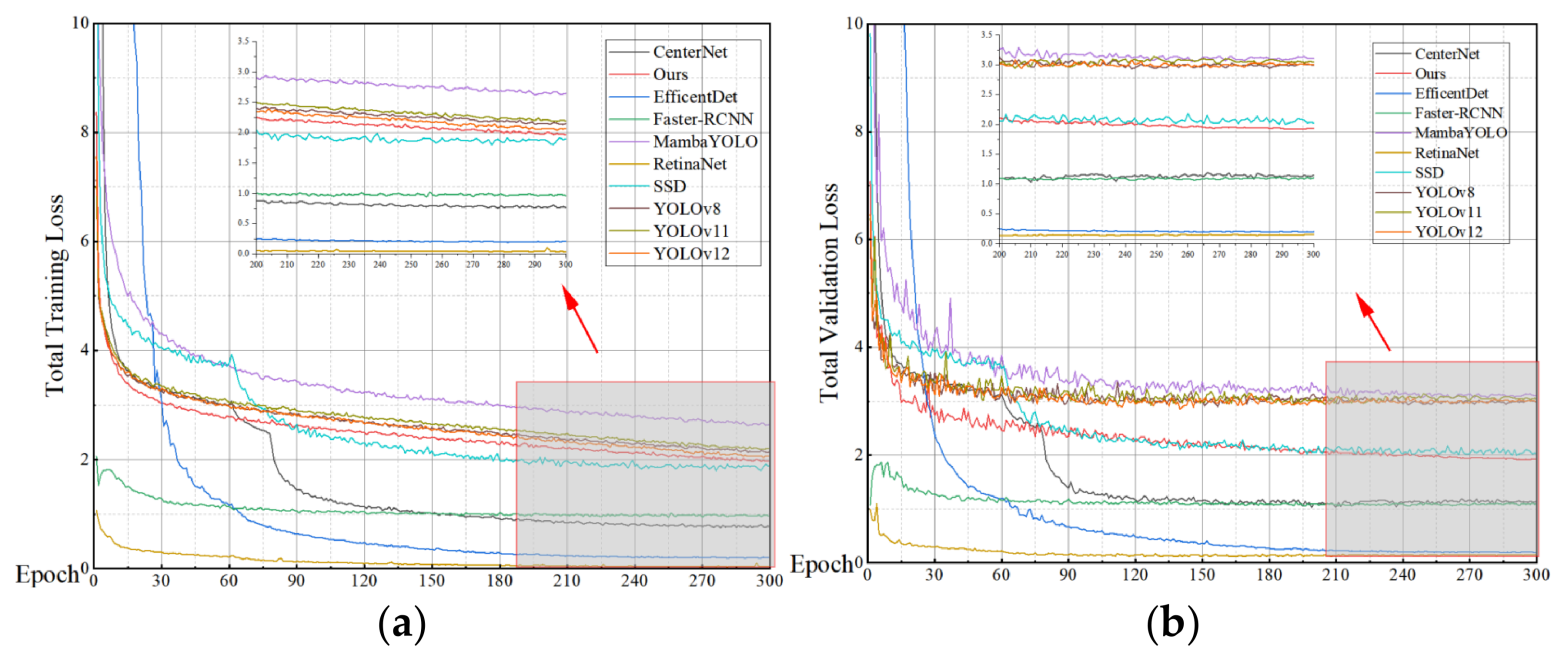
Training and validation loss curves of different detection models: (**a**) total training loss; (**b**) total validation loss. Different colors denote different detection models while the red arrows indicate regions selected for enlarged visualization.

To illustrate representative optimization behaviors, a subset of comparison models was selected for training and validation loss visualization. In addition to the quantitative comparison in Table 5, the optimization processes of different detection models were further analyzed using their total loss curves on the training and validation sets, as shown in Figure 12. Specifically, Figure 12a presents the training loss curves, whereas Figure 12b presents the validation loss curves. Compared with final detection metrics alone, these curves provide a more direct representation of model convergence and optimization stability during training.

As observed from Figure 12, most methods exhibit a rapid decrease in loss during the early training stage, indicating that the models can quickly learn the basic discriminative features of vibration damper targets. As training proceeds, differences among the models gradually emerge. Some comparative methods maintain relatively high loss values or exhibit evident fluctuations in the later training stage, indicating limited optimization stability in complex-background and small-object scenarios. In contrast, CARE-Net exhibits lower and more stable loss variation trends on both the training and validation sets, suggesting more favorable convergence behavior and generalization performance. This observation is consistent with the quantitative results reported in Table 5 and provides additional support for the effectiveness of the proposed framework.

### 3.5. Deployment-Oriented Visualization System

**Figure 13:**
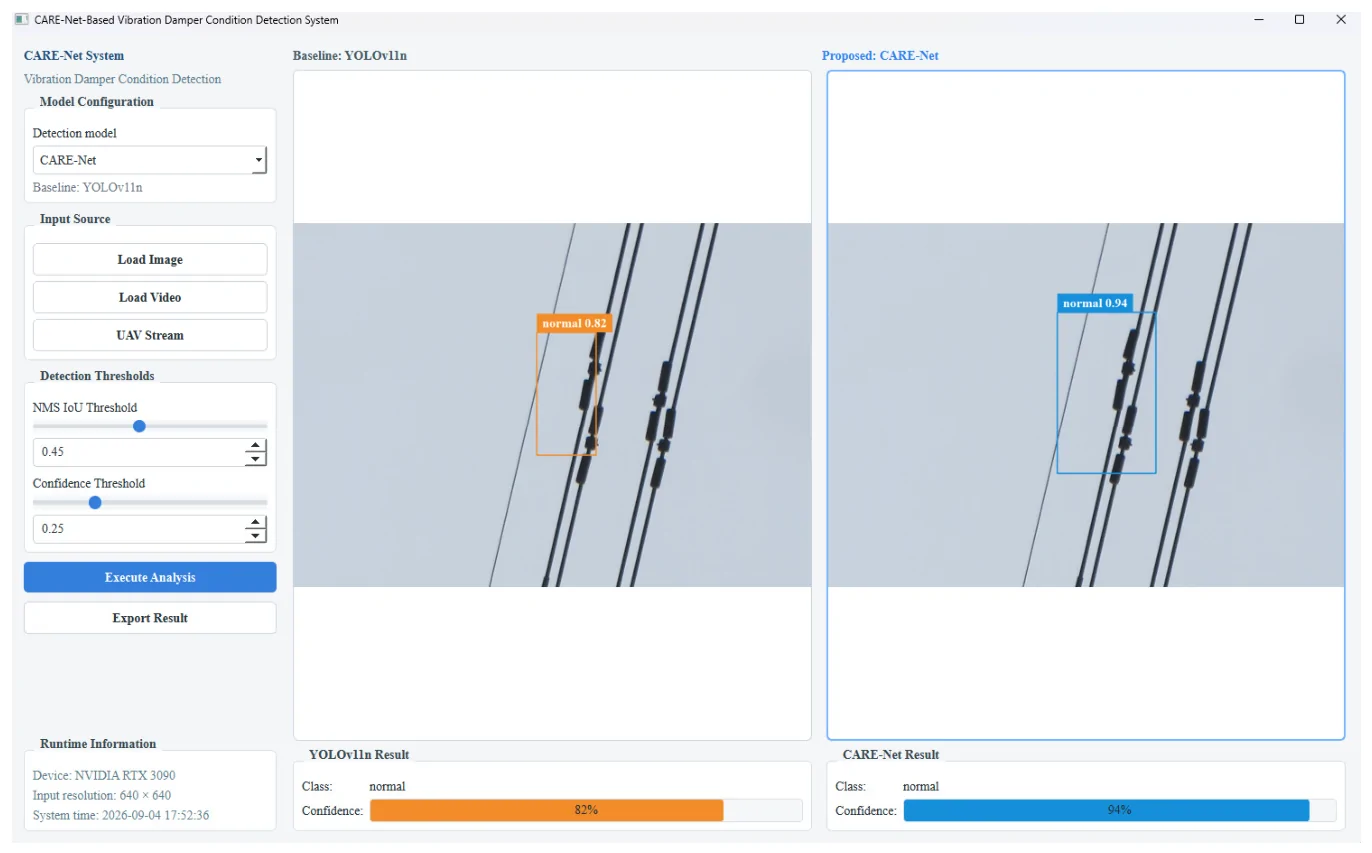
Visualization interface of the vibration damper detection system for transmission line inspection.

To further illustrate the potential integration of the proposed method into a practical inspection terminal, a visualization interface prototype for vibration damper detection was designed, as shown in Figure 13. The interface mainly consists of functional control, model selection, parameter setting, image display, and result output regions, and supports functions including image import, video input, camera access, detection execution, and result saving.

In the image display region, the left panel presents the detection results of the YOLOv11 baseline model, whereas the right panel presents the detection results of CARE-Net. Both results are generated from the same input image, thereby facilitating an intuitive comparison of the localization and recognition performance of different models for vibration damper targets. The information panel on the right displays key results, including the detection model, target category, confidence score, number of detected targets, and localization status. Through this interface, inspection personnel can rapidly examine vibration damper detection results and obtain the corresponding condition information, indicating the potential of CARE-Net for integration into intelligent transmission line inspection systems. Essentially, this interface is intended to illustrate the connection between model outputs and the inspection analysis workflow, thereby providing an interface design basis for subsequent software integration and operational validation on practical terminals.

## 4. Conclusions

The experimental results demonstrate that the key challenge in vibration damper detection does not arise solely from the small target scale. More importantly, the slender structural information of vibration damper targets is susceptible to degradation from background interference during feature propagation, while excessive attention enhancement in deep-level features may amplify semantic redundancy and disrupt cross-scale consistency. Based on this understanding, CARE-Net does not adopt a uniform enhancement strategy for features at all levels. Instead, structural enhancement and contextual refinement are deployed at different feature stages. CRAB preserves structural cues with discriminative value for edges, connecting parts, and overall contours at the front end, whereas DCRN dynamically refines contextual responses before deep feature fusion. The experiments on CRAB deployment position further reveal that, for such small and slender targets, the effectiveness of an enhancement mechanism depends not only on its form, but also on the feature level at which it is applied. The controlled comparisons with representative lightweight attention and dynamic-convolution configurations further support the suitability of CRAB and DCRN under compact model constraints.

Moreover, the ablation results reveal the dependency between regression optimization and feature representation. When introduced independently, AF-CIoU does not achieve the best overall performance, indicating that the effectiveness of quality-aware regression optimization is affected by the quality of front-end feature representation. Therefore, the performance improvement of CARE-Net results from the functional complementarity among different optimization stages: front-end structural enhancement preserves more discriminative contour information for slender targets, back-end contextual refinement improves the stability of semantic representation under complex backgrounds, and regression optimization further improves bounding-box localization quality with the support of the improved feature representation. This result indicates that, for slender and weak-texture targets in complex backgrounds, loss function design needs to be matched with feature representation capability in order to effectively improve detection stability and localization reliability. Finally, the comprehensive comparison results indicate that CARE-Net achieves competitive overall detection performance while maintaining a compact model scale and relatively low computational complexity.

This study further constructs a visualization interface connected with the inspection workflow to demonstrate how CARE-Net can be integrated into the terminal-side analysis stage. Future research will focus on larger and more diverse transmission-line inspection datasets, stricter cross-route and cross-instance evaluation, varying imaging conditions, and online inference and operational validation on edge devices, with the aim of further improving the generalization capability and deployment reliability of the model in long-term practical inspection tasks.

## Figures and Tables

**Table 1:** Quantitative ablation results under different module configurations.

CRAB	DCRN	AF-CIoU	Params	GFLOPs	Precision	Recall	mAP^@0.5^	mAP^@0.5:0.95^
			2.58 M	6.3 G	0.927	0.889	0.938	0.615
√			**2.44 M**	**6.2 G**	0.942	0.885	0.937	0.617
	√		2.58 M	6.3 G	0.951	0.911	0.933	0.616
		√	2.58 M	6.3 G	0.957	0.878	0.928	0.605
√	√		**2.44 M**	**6.2 G**	0.949	0.899	0.941	0.619
√	√	√	**2.44 M**	**6.2 G**	**0.967**	**0.922**	**0.951**	**0.628**

**Table 2:** Ablation study of CRAB placement at different feature level.

Backbone	P3	P4	P5	DCRN	P	R	mAP^@0.5^	mAP^@0.5:0.95^
√					0.942	0.885	0.937	0.617
√			√		0.949	0.891	0.931	0.611
√		√	√		0.939	0.876	0.923	0.615
√	√	√	√		0.395	0.463	0.402	0.192
√				√	0.949	**0.899**	**0.941**	**0.619**
√			√	√	0.918	0.894	0.911	0.602
√		√	√	√	0.938	0.863	0.926	0.612
√	√	√	√	√	**0.958**	0.842	0.913	0.592

**Table 3:** Comparison of CRAB and DCRN with representative alternative configurations.

Group	Module	Params	GFLOPs	mAP^@0.5^	mAP^@0.5:0.95^
Baseline	Original	2.58 M	6.3 G	0.938	0.615
CRAB Comparison	C3k2+ECA	2.58 M	6.3 G	0.939	0.614
	C3k2+CBAM	2.59 M	6.3 G	0.940	0.615
	CRAB	2.44 M	6.2 G	0.937	0.617
DCRN Comparison	C2PSA+ODConv	2.60 M	6.3 G	0.931	0.614
	DCRN	2.58 M	6.3 G	0.933	0.616

**Table 4:** Comparison of different bounding-box regression losses.

Loss Name	Precision	Recall	mAP^@0.5^	mAP^@0.5:0.95^
CIoU	0.949	0.899	0.941	0.619
EIoU	0.918	0.902	0.930	0.601
GIoU	0.935	0.897	0.935	0.608
ShapeIoU	0.954	0.918	0.945	0.610
SIoU	0.944	0.875	0.934	0.607
DIoU	**0.969**	0.877	0.936	0.607
AF-CIoU	0.967	**0.922**	**0.951**	**0.628**

**Table 5:** Comparison experiment of mainstream algorithms.

Model	Size	Params	GFLOPs	Precision	Recall	mAP^@0.5^	mAP^@0.5:0.95^
YOLOv8	640	3.01 M	8.1 G	0.951	0.902	0.937	0.603
YOLOv11	640	2.58 M	6.3 G	0.927	0.889	0.938	0.615
YOLOv12	640	5.99 M	13.6 G	0.931	0.897	0.930	0.598
Mamba-YOLO	640	5.98 M	13.6 G	0.931	0.873	0.928	0.595
Faster-RCNN	600	28.33 M	470.5 G	0.612	0.937	0.899	0.491
CenterNet	512	32.67 M	35.1 G	**0.973**	0.905	0.934	0.586
EfficientDet	512	3.83 M	**2.4 G**	0.898	0.642	0.767	0.453
RetinaNet	600	19.87 M	55.3 G	0.818	**0.948**	0.877	0.589
SSD	300	24.28 M	30.6 G	0.920	0.593	0.795	0.527
Hyper-YOLO-T	640	2.68 M	7.7 G	0.952	0.906	0.943	0.610
RT-DETR-R18	640	21.32 M	60.5 G	0.944	0.913	0.931	0.607
**Ours**	640	**2.44 M**	6.2 G	0.967	0.922	**0.951**	**0.628**

## Data Availability

The dataset and source code used in this study are not publicly available because the transmission-line inspection data are subject to data-use, privacy, and proprietary restrictions associated with the relevant collaborative projects. To facilitate reproducibility, detailed information regarding the dataset partition, training configurations, repeated-run settings, and evaluation protocol is provided in the manuscript. The data and source code may be made available by the corresponding author upon reasonable request, subject to permission from the relevant collaborative institutions.

## References

[1] Yang L., Fan J., Liu Y., Li E., Peng J., Liang Z. (2020). A Review on State-of-the-Art Power Line Inspection Techniques. IEEE Trans. Instrum. Meas..

[2] Faisal M.A.A., Mecheter I., Qiblawey Y., Hernandez-Fernandez J., Chowdhury M.E.H., Kiranyaz S. (2025). Deep learning in automated power line inspection: A review. Appl. Energy.

[3] Claren R., Diana G. (1969). Mathematical analysis of transmission line vibration. IEEE Trans. Power Appar. Syst..

[4] Sun Z., Wang X. (2025). Small target detection for power line components in complex UAV imagery. IEEE Access.

[5] Ahmed F., Mohanta J.C., Keshari A. (2024). Power transmission line inspections: Methods, challenges, current status and usage of unmanned aerial systems. J. Intell. Robot. Syst..

[6] Azevedo C.R.F., Henriques A.M.D., Pulino Filho A.R., Ferreira J.L.A., Araújo J.A. (2009). Fretting fatigue in overhead conductors: Rig design and failure analysis of a Grosbeak aluminium conductor steel-reinforced conductor. Eng. Fail. Anal..

[7] Li D., Wang X., Zhang J., Ji Z. (2023). Automated deep learning system for power line inspection image analysis and processing: Architecture and design issues. Glob. Energy Interconnect..

[8] Wu K., Chen Y., Lu Y., Yang Z., Yuan J., Zheng E. (2024). SOD-YOLO: A high-precision detection method for small targets on high-voltage transmission lines. Electronics.

[9] Song Z., Huang X., Ji C., Zhang Y. (2023). Deformable YOLOX: Detection and rust warning method of transmission line connection fittings based on image processing technology. IEEE Trans. Instrum. Meas..

[10] Yang K., Hu Z., Zhao X., Wang Q., Hu D., Zhai Y. (2025). One-for-all: Geometric structure-guided single-domain generalization for vibration damper defect detection. Eng. Appl. Artif. Intell..

[11] Zhao Z., Guo G., Pan Y., Zhang K., Zhai Y., Zhao W. (2025). Anti-vibration hammer defect detection based on structural knowledge representation. Eng. Appl. Artif. Intell..

[12] Liu J., Liu C., Wu Y., Sun Z. (2024). Recognition of vibration damper based on deep learning methods in UAV images. IEICE Trans. Inf. Syst..

[13] Wu Y., Chen E., Zheng L. (2023). Damper defect detection for transmission lines based on cognitive preprocessing and feature fusion. J. Electron. Imaging.

[14] Ji C., Zhang F., Huang X., Song Z., Hou W., Wang B., Chen G. (2024). STAE-YOLO: Intelligent detection algorithm for risk management of construction machinery intrusion on transmission lines based on visual perception. IET Gener. Transm. Distrib..

[15] Jia X., Ji C., Zhang F., Liu J., Gao M., Huang X. (2024). AdaptoMixNet: Detection of foreign objects on power transmission lines under severe weather conditions. J. Real-Time Image Process..

[16] Ji C., Gao M., Zhou S., Liu J., Zhu Y., Huang X. (2024). DRI-Net: A model for insulator defect detection on transmission lines in rainy backgrounds. J. Real-Time Image Process..

[17] Yi J., Mao J., Zhang H., Chen Y., Liu T., Zeng K., Xie H., Wang Y. (2025). Balancing accuracy and efficiency with a multiscale uncertainty-aware knowledge-based network for transmission line inspection. IEEE Trans. Ind. Informat..

[18] Zheng Z., Ye R., Wang P., Ren D., Zuo W., Hou Q., Cheng M.-M. Localization distillation for dense object detection. Proceedings of the 2022 IEEE/CVF Conference on Computer Vision and Pattern Recognition (CVPR).

[19] Li X., Wang W., Wu L., Chen S., Hu X., Li J., Tang J., Yang J. (2020). Generalized focal loss: Learning qualified and distributed bounding boxes for dense object detection. Adv. Neural Inf. Process. Syst..

[20] Zhang Y., Yuan X., Li W., Chen S. (2017). Automatic power line inspection using UAV images. Remote Sens..

[21] Zhu Y., Huang X., Zhang G., Zhao L., Tian Y. (2018). Application research of the power supply for transmission line on-line monitoring devices. High Volt. Appar..

[22] Shi W., Cao J., Zhang Q., Li Y., Xu L. (2016). Edge computing: Vision and challenges. IEEE Internet Things J..

[23] Wei S., Cai Y., Dong K., Liu C., Yu F., Zhong S. (2026). Towards autonomous powerline inspection: A real-time UAV-edge computing framework for early identification of fire-related hazards. Drones.

[24] Yan Z., Chen C., Wu S., Wang Z., Li L., Sun S., Yang B., Fu J. (2025). RF-DET: Refocusing on the small-scale objects using aggregated context for accurate power transmitting components detection on UAV oblique imagery. ISPRS J. Photogramm. Remote Sens..

[25] Martinez C., Sampedro C., Chauhan A., Collumeau J.F., Campoy P. (2018). The Power Line Inspection Software (PoLIS): A versatile system for automating power line inspection. Eng. Appl. Artif. Intell..

[26] Luo Y., Yu X., Yang D., Zhou B. (2023). A survey of intelligent transmission line inspection based on unmanned aerial vehicle. Artif. Intell. Rev..

[27] Zhao Z., Zheng P., Xu S., Wu X. (2019). Object detection with deep learning: A review. IEEE Trans. Neural Netw. Learn. Syst..

[28] Zhang J., Li X., Li J., Liu L., Xue Z., Zhang B., Jiang Z., Huang T., Wang Y., Wang C. Rethinking mobile block for efficient attention-based models. Proceedings of the IEEE/CVF International Conference on Computer Vision.

[29] Liu X., Peng H., Zheng N., Yang Y., Hu H., Yuan Y. EfficientViT: Memory-efficient vision transformer with cascaded group attention. Proceedings of the IEEE/CVF Conference on Computer Vision and Pattern Recognition.

[30] Zhu J., Chen X., He K., LeCun Y., Liu Z. (2025). Transformers without normalization. arXiv.

[31] Zheng Z., Wang P., Liu W., Li J., Ye R., Ren D. (2020). Distance-IoU loss: Faster and better learning for bounding box regression. Proc. AAAI Conf. Artif. Intell..

[32] Zhang Y.-F., Ren W., Zhang Z., Jia Z., Wang L., Tan T. (2022). Focal and Efficient IoU Loss for Accurate Bounding Box Regression. Neurocomputing.

[33] Tian Y., Ye Q., Doermann D. (2025). YOLOv12: Attention-centric real-time object detectors. arXiv.

[34] Wang Z., Li C., Xu H., Zhu X., Li H. (2024). MambaYOLO: A simple baseline for object detection with state space model. arXiv.

[35] Liu W., Anguelov D., Erhan D., Szegedy C., Reed S., Fu C.Y., Berg A.C. (2016). SSD: Single shot multibox detector. Proceedings of the European Conference on Computer Vision.

[36] Ren S., He K., Girshick R., Sun J. (2017). Faster R-CNN: Towards real-time object detection with region proposal networks. IEEE Trans. Pattern Anal. Mach. Intell..

[37] Duan K., Bai S., Xie L., Qi H., Huang Q., Tian Q. CenterNet: Keypoint triplets for object detection. Proceedings of the IEEE/CVF International Conference on Computer Vision.

[38] Tan M., Pang R., Le Q.V. EfficientDet: Scalable and efficient object detection. Proceedings of the IEEE/CVF Conference on Computer Vision and Pattern Recognition.

[39] Lin T.Y., Goyal P., Girshick R., He K., Dollár P. (2020). Focal loss for dense object detection. IEEE Trans. Pattern Anal. Mach. Intell..

[40] Feng Y., Huang J., Du S., Ying S., Yong J.H., Li Y., Ding G., Ji R., Gao Y. (2024). Hyper-YOLO: When visual object detection meets hypergraph computation. arXiv.

[41] Zhao Y., Lv W., Xu S., Wei J., Wang G., Dang Q., Liu Y., Chen J. DETRs beat YOLOs on real-time object detection. Proceedings of the IEEE/CVF Conference on Computer Vision and Pattern Recognition.

